# Occupational radiation safety and sustainable workforce management in PET/CT practice: a pilot study of healthcare professionals in a newly established cyclotron-based nuclear medicine center

**DOI:** 10.3389/fpubh.2026.1782228

**Published:** 2026-03-27

**Authors:** Suphalak Khamruang Marshall, Kunlagan Mewes, Nadia Noomad, Wanita Durawee, Awatif Hayeeabdunromae

**Affiliations:** 1 Department of Radiology, Faculty of Medicine, Prince of Songkla University, Songkhla, Thailand; 2 Department of Business Administration, Faculty of Management Sciences, Prince of Songkla University, Songkhla, Thailand

**Keywords:** ALARA principle, extremity dosimetry, occupational radiation exposure, PET/CT nuclear medicine, quality of work life, radiation protection management, workforce sustainability

## Abstract

**Background:**

The working environment of nuclear medicine staff is a critical factor in occupational sustainability, particularly in newly established clinical services where safety systems and workflows are still developing.

**Objectives:**

This study examined the relationship between quality of work life (QWL), job satisfaction, and occupational radiation exposure among healthcare professionals in a newly established cyclotron and molecular imaging nuclear medicine center equipped with a PET/CT facility that has been operational for less than 5 years.

**Methods:**

A mixed-methods design was applied, integrating a cross-sectional assessment of QWL and job satisfaction with environmental radiation monitoring and personal dose mapping associated with ^18^F-FDG procedures. Participants included radiochemists, nurses, radiological technologists, and support staff.

**Results:**

Qualitative findings indicated strong theoretical knowledge of radiation protection principles following structured training; however, early-stage operational barriers such as limited access to protective equipment, lack of real-time dosimetry, and workflow pressures often hindered consistent safety compliance. These issues motivated a quantitative evaluation of occupational dose distribution across different body regions. Environmental dose mapping and extremity dosimetry revealed significant variation in exposure among professional groups, with radiochemists demonstrating the highest extremity doses, particularly at the right thumb, index finger, and palm (*p* < 0.05), reflecting direct manual handling during radiopharmaceutical synthesis and dispensing. Despite moderate overall job satisfaction, safety training improved radiation safety awareness by approximately 80% and increased adherence to radiation protection protocols from 14.29% to 87%.

**Conclusion:**

The findings suggest that while education may enhance radiation safety awareness, early-stage workflow constraints and manual handling tasks may remain important contributors to occupational exposure. These results emphasize the potential importance of integrating accurate dose monitoring, ergonomic workflow design, and continuous education during the early development of nuclear medicine services to help maintain exposures within ALARA limits and support a resilient and sustainable healthcare workforce. Furthermore, the findings indicate that combining objective radiation monitoring with organizational and psychosocial assessment may help inform proactive occupational health planning in newly established nuclear medicine centers.

## 1 Introduction

In the occupational environment, radiation detection and radiation protection have proven to be a key factor in how healthcare professionals evaluate their quality of work life (QWL) and are a crucial determinant of the quality and safety of patient care (1, 2). In nuclear medicine, this connection feels even stronger. As medical workers in the field of nuclear medicine on a day-to-day basis deal with complicated diagnostics, work under immense pressure, and carry the mental load of working around radiation, even if the risks are controlled and well understood (3). During the COVID-19 pandemic, the importance of QWL of medical professionals came into the spotlight (4). However, it is well reported about the concerns of healthcare professionals’ well-being long before COVID arrived, but the sheer pressure caused by the pandemic made QWL a factor impossible to ignore. Moreover, in difficult times, QWL becomes about more than just avoiding burnout; it is about keeping people motivated and engaged (5, 6). More recently, a study by Smith-Bindman et al. predicted that the current U. S. CT use (≈93 million tests in 2023) might cause ~103,000 malignancies (~5% of annual diagnoses) (7). Resulting in mass media coverage that has indirectly had a detrimental influence on nuclear medical professionals. Patients experiencing elevated anxiety levels prior to CT scans necessitate additional time for counseling and obtaining patient permission, which in turn causes delays or even cancelations of the procedure.

Furthermore, a number of factors contribute to shaping QWL and influencing employees’ work experiences. Factors such as job satisfaction, the working environment, access to training, career advancement opportunities, how people get along with colleagues, and how safe or secure they feel in their roles (8). Noteworthy, a correlation was found between QWL activities and great staff performance and morale (9, 10). However, organizations suffer if staff are unable to accomplish a good balance between their work and the rest of their lives. Characterized by disengagement and reduced productivity, people check out mentally despite remaining in their roles; they reduce their work contributions and disengage from their organization This disengagement has garnered considerable attention, particularly in relation to what is now known as “quiet quitting.” In healthcare, this could appear as individuals adhering strictly to the fundamental aspects of their roles without engaging in additional responsibilities. Moreover, the escalating trend of quiet quitting highlights the unrelenting challenge of sustaining a motivated and engaged workforce. Therefore, it is essential to acknowledge these influencing factors in order to gain a comprehensive understanding of the working environment in which our research is conducted (11, 12). These challenges represent critical determinants of the working environment that directly influence staff performance, well-being, and safety. The present research aims to systematically identify, analyze, and interpret these issues, elucidate their underlying causes, assess their implications for professional practice, and provide an evidence-based foundation for potential interventions.

In particular, to achieve balance between job satisfaction and safety concerns in nuclear medicine departments makes this issue even more complex. Departments in this field are characterized by sophisticated technology, advanced technical expertise, and strict radiation protocols, which create a complex working environment for studying (13, 14). With increasing occupational demands in nuclear medicine, accurate radiation detection and dose assessment are essential to ensure worker safety, regulatory compliance, and long-term workforce sustainability. This study addresses key dimensions of QWL—including occupational health and safety, training, workload, and organizational support—while focusing primarily on the objective evaluation of occupational radiation exposure. In response to staff concerns regarding radiation risk, a mixed-methods approach was implemented that integrates qualitative assessment of radiation-safety perceptions with quantitative environmental radiation monitoring and personal dosimetry in a nuclear medicine department housing PET/CT radiopharmaceutical preparation, dose administration, and patient waiting areas. Radiation exposure from ^18^F-FDG procedures was quantified using area dose rate measurements, whole-body effective dose, and extremity equivalent dose, consistent with the radiological protection framework defined by ICRP Publication 103 and IAEA General Safety Requirements Part 3. The primary objective was to determine whether perceived radiation-safety practices were supported by measured occupational dose distributions and to identify task-specific exposure patterns, particularly those associated with manual radiopharmaceutical handling. By combining environmental dose mapping with extremity and personal dosimetry, this study provides a comprehensive evaluation of occupational radiation risk and safety culture, supporting the development of system-level radiation protection strategies that integrate technical controls, organizational measures, and continuous education to maintain exposures within ALARA (As Low As Reasonably Achievable) limits, with particular emphasis on high-risk roles such as radiochemists and nursing staff in a newly established cyclotron and molecular imaging nuclear medicine center (<5 years).

## 2 Materials and methods

### 2.1 Study design and survey distribution

The primary goal of this study is to investigate and ascertain any relationships between the QWL and job satisfaction of nuclear medicine staff at Songklanagarind Hospital in Thailand. Therefore, a cross-sectional survey was used to collect data, with the key objective of identifying specific links and correlations among variables and evaluating the prevalence of staff behaviors and beliefs. Accordingly, to accomplish this, a structured questionnaire was designed as the major data gathering tool. Therefore, to verify the questionnaire’s dependability, a thorough review of relevant literature was conducted prior to questionnaire development, including pre-existing, validated scales. Furthermore, combining closed- and open-ended questions allows for a more comprehensive understanding of participants’ perspectives and experiences.

The cohort study included all radiation professionals categorized according to work characteristics and functional roles across four occupational groups at a newly established cyclotron and molecular imaging nuclear medicine center (<5 years), Songklanagarind Hospital, Thailand. The survey was administered to healthcare personnel within this center during its early operational phase (*n* = 20), including radiochemists (*n* = 4), nurses (*n* = 4), radiological technologists (*n* = 5), and radiological technologist assistants (*n* = 7). To optimize response rates and ensure data accuracy, the survey was distributed using a combination of electronic and paper-based methods (15). The survey was distributed using both electronic and paper-based formats to optimize accessibility and response rates. Reminder notifications were issued 2 weeks after initial distribution, and participants were given 2 weeks to complete the questionnaire. All responses were anonymized and securely stored.

### 2.2 Psychometric validation and reliability assessment

The QWL instrument was adapted from previously validated occupational health and workforce well-being scales reported in the literature (16–21). The selection of items was guided by established theoretical frameworks addressing work-related stress, organizational support, emotional exhaustion, and job satisfaction among healthcare professionals. Appropriate references have been added to support the conceptual validity of the instrument. To ensure contextual suitability for the Thai healthcare setting, a structured cultural adaptation process was conducted. This included forward translation by bilingual experts, independent back-translation to confirm semantic equivalence, and review by a multidisciplinary expert panel (comprising a medical physicist, occupational health specialist, and senior nuclear medicine practitioner) to evaluate content validity, clarity, and contextual appropriateness. Minor wording refinements were implemented to enhance comprehension while preserving conceptual integrity.

A pilot test was conducted with a small group of healthcare personnel not included in the final analysis to assess feasibility, clarity, and item relevance. Feedback from the pilot phase informed minor revisions prior to final administration. Internal consistency reliability was assessed using Cronbach’s alpha coefficients to evaluate the degree to which items within each scale measured a coherent construct. Cronbach’s alpha values ≥ 0.70 were considered indicative of acceptable reliability, consistent with established psychometric standards. Although the sample size was limited, reliability testing was conducted to ensure measurement stability and internal coherence of the instrument prior to inferential analysis.

### 2.3 Qualitative study on the perspectives and training needs of nuclear medicine personnel

This qualitative study aims to explore nuclear medicine staff perspectives on radiation-related work, focusing specifically on staff training needs and their QWL. The study design is guided by the Consolidated Criteria for Reporting Qualitative Research (COREQ), ensuring a comprehensive and structured approach to conducting and reporting interviews and focus group data (22). The study utilized thematic analysis to identify recurring patterns and themes within the data. Healthcare personnel in a newly established cyclotron and molecular imaging nuclear medicine center were invited to participate and encouraged to participate, enabling a diverse yet focused group. Face-to-face interviews, for a period of between 25 and 30 min each, and performed using a semi-structured guide to ensure consistency while allowing an in-depth exploration of the participants’ responses. Moreover, the guide covered topics such as perceptions of radiation protection training, benefits, challenges, and staff suggestions for improvement. Open-ended questions and probing encouraged detailed insights. All of the staff one to one interviews were accurately transcribed, followed by thematic analysis, compromising coding, pattern identification, and theme categorization, with direct quotations highlighting all key findings (23, 24). Additionally, the Office of Human Research Ethics Committee, Faculty of Medicine, Prince of Songkla University, Thailand granted ethical approval (REC. 66–376–7-2, 28 November 2023) for the study. Additionally, informed consent was given by all of the staff participating in this study, and in compliance with guidelines provided by the Declaration of Helsinki and the International Conference on Harmonization for Good Clinical Practice (ICH-GCP).

### 2.4 Radiation safety training needs assessment and program implementation

**Figure 1:**
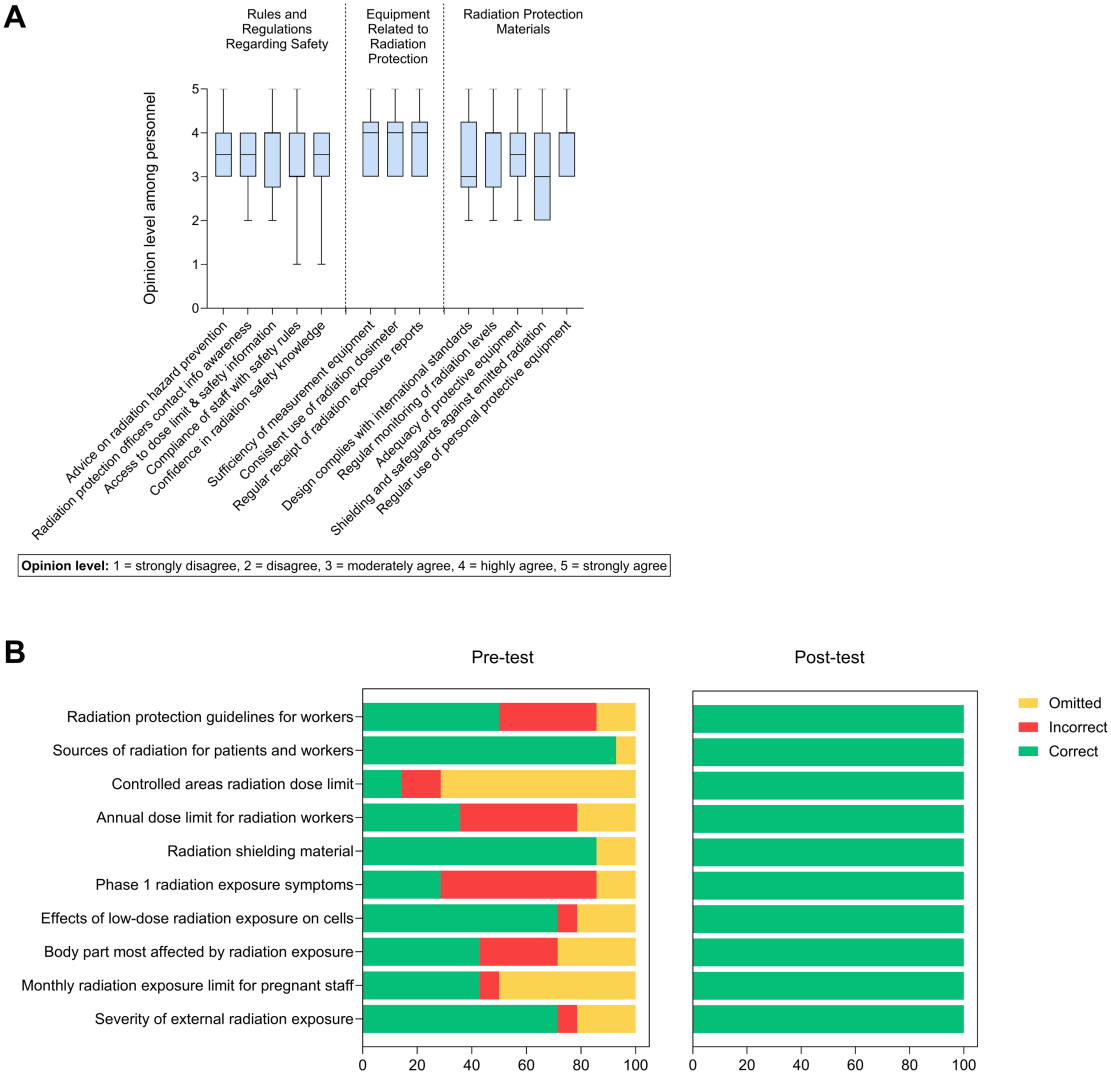
Assessment of radiation knowledge and protection practices in nuclear medicine staff. (**A**) Boxplot of basic knowledge of radiation and protection among nuclear medicine personnel (*n* = 20), categorized into safety regulations, protective equipment, and shielding materials. Responses were rated on a 5-point Likert scale (1 = strongly disagree to 5 = strongly agree). Median, interquartile range, and data range are shown; values are expressed as mean ± standard deviation. (**B**) The comparison of response accuracy for 10 radiation-safety knowledge questions administered before (pre-test) and after (post-test) the training intervention. Each horizontal bar represents a single knowledge domain related to radiation protection, including dose limits, shielding principles, biological effects of radiation, and regulatory guidelines. Responses are categorized as correct, incorrect, or omitted, and expressed as percentages of total respondents. Color intensity reflects response frequency, with darker shading indicating a higher proportion of responses within each category.

A structured training needs assessment based on internal safety audits and staff feedback identified gaps in radiation protection knowledge and workflow compliance, informing development of a targeted radiation safety training program. The program was implemented during the early operational phase of the cyclotron-based PET/CT center, with all occupational groups invited to participate and attendance documented. Training was delivered onsite through lectures, workshops, and practical demonstrations covering radiation protection principles, ALARA application, extremity dosimetry, shielding optimization, radiopharmaceutical handling, workflow standardization, and emergency procedures. The training was structured into three sessions, each lasting 2 hrs. Sessions were led by a certified medical physicist and the departmental Radiation Safety Officer. Effectiveness was evaluated using structured pre- and post-training assessments and compliance observations, with pre–post differences analyzed using non-parametric methods (Figure 1; Supplementary Table S1). A detailed description is provided in the Supplementary material.

### 2.5 Assessment of environmental radiation exposure in ^18^F-FDG workplace

As a result of feedback from the qualitative study and concerns about radiation exposure, an environmental radiation assessment was conducted in a center, which houses the PET/CT facility’s radiopharmaceutical preparation area, dose administration area, and patient waiting zones. The architectural layout comprised six functional zones: (i) hot waiting area adjacent to the molecular imaging and cyclotron center (ii) patient registration/screening, (iii) entrance to the PET/CT scanner, (iv) control room entrance, (v) control/monitoring zone, and (vi) PET/CT gantry (primary exposure zone). The ambient dose rate measurements were quantified using a calibrated portable survey meter Geiger counter (Ludlum Measurements, Inc., Sweetwater, TX, United States), which can detect photon energies in the range of 30 keV to 1.3 MeV. The device was calibrated at an accredited secondary standard dosimetry laboratory prior to use, ensuring measurement traceability to national standards. The detector response was verified daily using a ^137^Cs reference source to confirm stability within ±5%. Measurements were performed during routine ^18^F-FDG patient studies under normal operational conditions. The survey meter was positioned at a height of 1.2 m above the floor level to simulate occupational staff torso level. Dose rates were recorded at grid points spaced at 1.0 m intervals throughout the facility. In each zone, measurements were taken during active radiopharmaceutical handling, injection, and image acquisition phases to enable the capture of any maximum exposure levels. Each measurement was repeated three times to provide a statistical estimate of variability and reliability, and the mean value was applied for subsequent analysis. The resulting heatmaps were color-coded on a logarithmic scale ranging from 0.1 μSv/h (background) to 10 μSv/h (high-exposure areas). The heatmaps were overlaid onto the architectural floor plan to enable visualization of the radiation intensity gradients across all nuclear medicine work zones. Moreover, all the measurements were performed in compliance with IAEA Safety Standards Series No. GSG-17 (25) and GSG-18 (26) (superseding RS-G-1.1) and ICRP Publication 103 recommendations for occupational exposure. Throughout the survey, staff activities were observed concurrently in order to correlate dose variations with individuals’ operational behavior and movement patterns. In addition, all radiation warning signs and controlled-area boundaries were verified to comply with international regulatory requirements to ensure accurate zone classification.

### 2.6 Evaluation of occupational exposure assessment of healthcare personnel during ^18^F-FDG procedures

The objective of this study was to address safety concerns of medical staff working in the center and to determine staff occupational radiation exposure from ^18^F-FDG procedures. The radiochemists primary responsibility being radiopharmaceutical synthesis and dispensing, whereas nurse’s role being intravenous ^18^F-FDG administration and patient care during tracer uptake. The role of the radiological technologists to operate the PET/CT scanner and manage image acquisition, and radiological technologist assistants facilitate patient transfer. All participants in the study were full-time employees with at least one-year clinical experience. Additionally, to quantify both whole-body and extremity doses staff personal radiation monitoring was implemented using nanoDot small-type optically stimulated luminescence (OSL) dosimeters and Quixel OSL badges (Landauer Inc., Glenwood, IL, United States) (27). Throughout radiopharmaceutical preparation, dose dispensing, patient injection, and image acquisition dosimeters were worn by all participants. The mean administered activity per patient ranged between 370 and 555 MBq of ^18^F-FDG. All whole-body and extremity dose measurements were collected over a period of 1 month, representing standard operational conditions and workload distribution. All individual exposure times and number of patient interactions were logged daily in order to normalize measured doses per working day exposure. The nuclear medicine facility was equipped with standard radiation protection measures, which include hot cell lead-shielding, tungsten syringe shields, and remote handling devices. Additionally, measured doses were expressed in micro Sieverts (μSv) and normalized per working day. The effective dose (E) was estimated according to tissue-weighting factors specified in ICRP Publication 103 (28). All assessments adhered to the principles of occupational radiation safety, emphasizing dose optimization under the ALARA framework.

### 2.7 Data analysis

Descriptive statistics were calculated for each survey item, including frequencies and percentages of responses. Group comparisons across the four occupational categories (radiochemists, nurses, radiological technologists, and radiological technologist assistants) were conducted using non-parametric methods. Differences in questionnaire responses were evaluated using the Kruskal–Wallis one-way analysis of variance, with statistical significance set at *p* ≤ 0.05 (29). Chi-square tests were applied to examine associations between demographic and occupational variables.

Normality was assessed using the Shapiro–Wilk test. Given the small sample size (*n* = 20) and the ordinal nature of psychosocial scales, non-parametric approaches were adopted. Associations between radiation dose metrics (extremity and whole-body dose) and psychosocial outcomes (QWL composite score and job satisfaction) were evaluated using Spearman’s rank correlation (*ρ*) with bootstrap-derived 95% confidence intervals. To account for potential occupational clustering, partial Spearman correlations controlling for occupational group were conducted as sensitivity analyses. Multivariable regression was not used as a primary approach due to the limited sample size relative to the number of parameters required for group adjustment and the risk of overfitting; regression results are therefore presented as exploratory in Supplementary material.

To further address clustering and distributional considerations, a rank-based ANCOVA was performed by rank-transforming both QWL and dose variables and fitting an OLS model of rank (QWL) ~ rank(dose) + occupational group. Additional sensitivity analyses included group-adjusted linear regression with HC3 robust standard errors and stratified bootstrap 95% confidence intervals generated by resampling within occupational groups. Because job satisfaction exhibited only two observed levels (score 3 vs. 4), associations were additionally examined using L2-regularized logistic regression with occupational group adjustment. All inferential analyses were interpreted as exploratory of the pilot sample size.

## 3 Results

### 3.1 Demographic and radiation work-related characteristics of the study group

This study surveyed 20 healthcare personnel in the center, most of whom were female (85.71%) and aged 30–39 years (42.86%). The majority held a bachelor’s degree (71.43%) and worked as radiochemists (20%), radiological technologists (25%), and nurses (20%). Most had 1–5 years of experience (71.43%), worked 40–50 h per week (85.71%), and abstained from smoking and alcohol. Despite working in a high-risk environment, only 14.29% of participants reported consistently following radiation protection procedures. Furthermore, 64.29% had never received formal radiation safety training, and only two individuals held the Radiation Safety Officer (RSO) license. However, 78.57% expressed a desire for annual hands-on training. Table 1 Chi-square (*χ*^2^) test results provide a good analysis of the relationships between categorical variables. Chi-square (*χ*^2^) reveal no statistically significant associations between gender, education level, exercise habits, or job position and radiation training, RSO certification, or concern about radiation risks (all *p* > 0.5). Furthermore, the results suggest limited variation in radiation safety behaviors across demographic and job-related factors within this sample.

**Table 1:** Chi-square test results assessing the association between selected demographic, occupational, and behavioral variables and radiation protection training, certification, and risk perception among radiation professionals (*n* = 20).

Criteria	Chi-square	*p*-value	Interpretation
Gender vs. Training	0.12	0.73	Not significant
Education vs. RSO license	0.43	0.81	Not significant
Exercise vs. Concern level	2.67	0.62	Not significant
Working position vs. Radiation training	2.23	0.53	Not significant

Overall, the results indicate a clear need for improved and consistent radiation protection training across all professional roles within nuclear medicine. Although no statistically significant differences were observed among demographic or occupational groups, the low rates of training and certification highlight the necessity of implementing structured and continuous safety education programs. In many countries, nuclear medicine personnel are required to undergo extensive radiation protection training to safeguard patients, colleagues, and the broader public. International regulatory bodies, including the Nuclear Regulatory Commission (NRC) and the International Commission on Radiological Protection (ICRP), provide comprehensive guidelines on radiopharmaceutical safety, which should serve as the foundation for institutional training and certification initiatives (30).

### 3.2 Basic radiation knowledge and protection from radiation hazards

This investigation explored both the perceived and actual knowledge of radiation safety among nuclear medicine personnel, with a focus on three primary domains: regulatory compliance, equipment usage, and material-based protective measures. Data was collected using Likert-scale opinion ratings (Figure 1A) alongside objective, multiple-choice knowledge assessments (Figure 1B), providing a multidimensional profile of radiation safety competencies. The boxplot in Figure 1A presents the self-reported perceptions of personnel. Overall, responses clustered around moderate agreement (Likert 3–4), indicating general but not unanimous confidence in radiation protection practices. Notably, greater variability was observed in areas concerning the use of personal protective equipment and shielding methods, suggesting inconsistent understanding or implementation. These findings emphasize the importance of reinforcing both theoretical knowledge and practical application in targeted training programs to ensure comprehensive radiation safety competence. The participants generally expressed favorable perceptions regarding their radiation safety practices. Noteworthy, the lack of a high level of agreement in the domain of equipment-related protection reflects an inadequate adherence to routine use of personal dosimeters, consistent radiation monitoring, and systematic reporting. Additionally, the score regarding shielding and safeguards had the lowest perception score, which suggests the insufficient availability of shielding and safeguards. These findings suggest a review of the availability of safety equipment and protocols should be carried out. To ensure staff are sufficiently shielded and compliance with radiation monitoring protocols is firmly embedded in day-to-day operations, and audited on a regular basis.

Figure 1B presents response accuracy for 10 radiation protection knowledge items. While general safety principles were well understood, deficiencies were observed in occupational dose limits, biological effects of low-dose exposure, and shielding properties. Although 71.34% correctly identified the risks of external radiation exposure, 21.34% provided no response and 7.14% answered incorrectly. High rates of incorrect or omitted responses were also noted for questions regarding pregnancy-related risks and early radiation syndrome, indicating clinically relevant knowledge gaps.

After completion of the structured radiation safety training, all participants achieved ≥85% on the competency assessment and 100% on the written examination, meeting the threshold for independent practice. The elimination of omitted responses in the post-test, together with improved response accuracy, demonstrates the effectiveness of the structured training program. These findings highlight the importance of continuous, competency-based education in strengthening radiation protection practices and sustaining a robust safety culture in nuclear medicine.

### 3.3 Opinions and satisfaction with the quality of work life among nuclear medicine personnel

**Figure 2:**
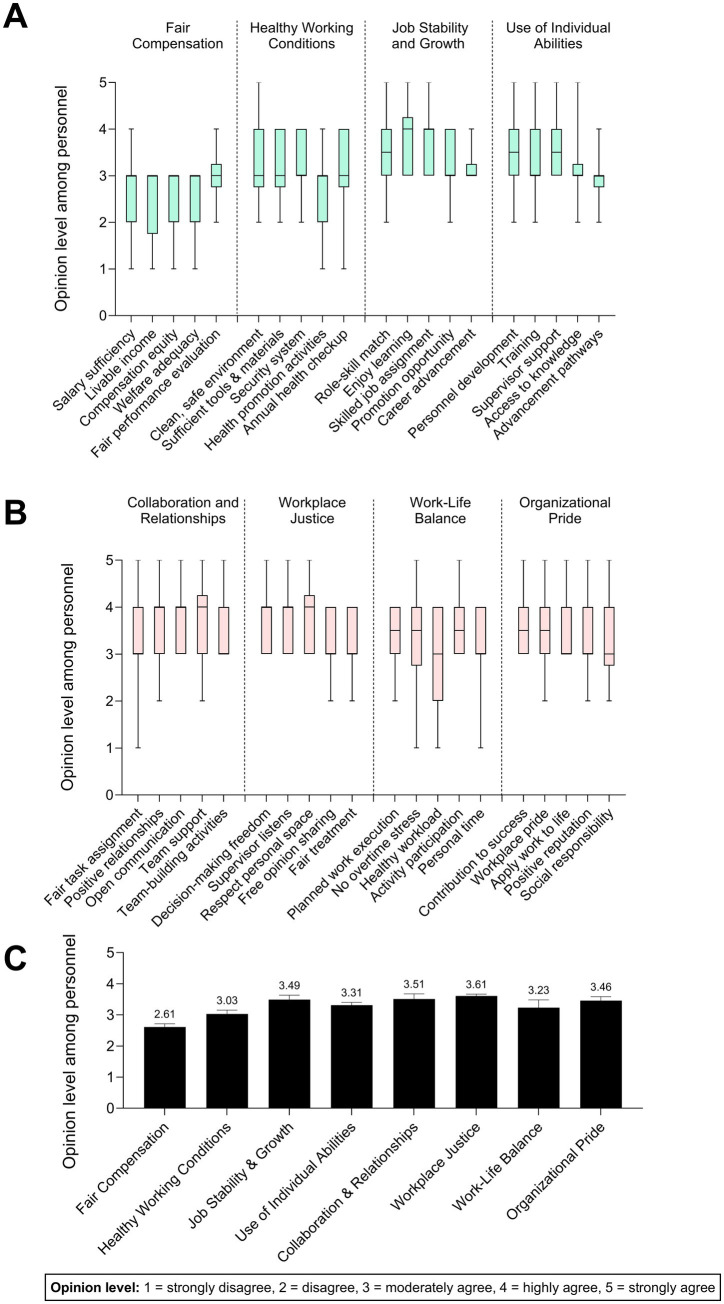
Distribution of opinion levels among nuclear medicine personnel (*n* = 20) across eight domains of job satisfaction. Boxplots depict self-reported ratings on a 5-point Likert scale, where 1 = strongly disagree and 5 = strongly agree. **(A)** Perceptions related to fair compensation, healthy working conditions, job stability and growth, and use of individual abilities. **(B)** Perceptions concerning collaboration and relationships, workplace justice, work-life balance, and organizational pride. Each domain consists of five representative indicators reflecting specific aspects of workplace quality and employee experience. Median, interquartile range, and whiskers indicate variability in perceived satisfaction levels. **(C)** Comparison of average opinion scores (mean ± standard deviation) across all eight dimensions.

QWL was assessed across eight domains reflecting remuneration, working conditions, professional development, organizational climate, and work-life balance. Domain-level mean scores and standard deviations are presented in Figure 2. Fair compensation demonstrated the lowest overall mean, with livable income receiving the lowest domain score, indicating central tendency toward lower satisfaction categories. Fair pay relative to peers similarly reflected reduced mean values. Standard deviations within compensation-related items were comparatively larger, indicating greater response variability and heterogeneous perceptions among staff. In contrast, fair performance evaluation showed relatively higher mean values with narrower dispersion, suggesting more consistent agreement. Healthy working conditions suggested moderate mean scores with limited variability, indicating neutral-to-slightly positive perceptions. Adequacy of tools and materials was similarly positioned within mid-range categories. Health promotion activities received comparatively lower ratings, suggesting weaker institutional emphasis on preventive staff wellness. Job security yielded moderate mean values, indicating neither pronounced insecurity nor strong confidence in stability. Domains related to skilled job assignment, learning alignment, and training opportunities received comparatively higher mean scores with modest dispersion, positioning professional development as a relative strength within the QWL structure. Additionally, Figure 2B illustrates collaboration, workplace justice, work-life balance, and organizational pride domains. Workplace justice demonstrated relatively high mean values and low standard deviations, indicating favorable and consistent perceptions. In contrast, work-life balance—particularly the healthy workload item—showed lower mean values with comparatively higher dispersion, suggesting uneven workload experiences across respondents. Open opinion sharing also scored lower, indicating potential communication constraints (31, 32). Figure 2C summarizes domain-level means with error bars representing standard deviations. Domains such as workplace justice and collaboration exhibited tighter clustering (lower SD), whereas compensation and work-life balance showed broader dispersion, reflecting greater heterogeneity in staff experience.

Importantly, internal consistency reliability for the QWL instrument was acceptable (Cronbach’s *α* ≥ 0.70 across domains), supporting the coherence of domain-level aggregation and providing reasonable confidence in the observed distributional patterns within this sample. Overall, the data indicate moderate overall QWL with domain-specific imbalances, particularly in compensation and workload dimensions. Although exploratory, these findings raise concerns regarding workload and compensation pressures in the early operational phase of the center and highlight the importance of contextual interpretation.

### 3.4 Tendency to continue working at the hospital among nuclear medicine personnel

**Figure 3:**
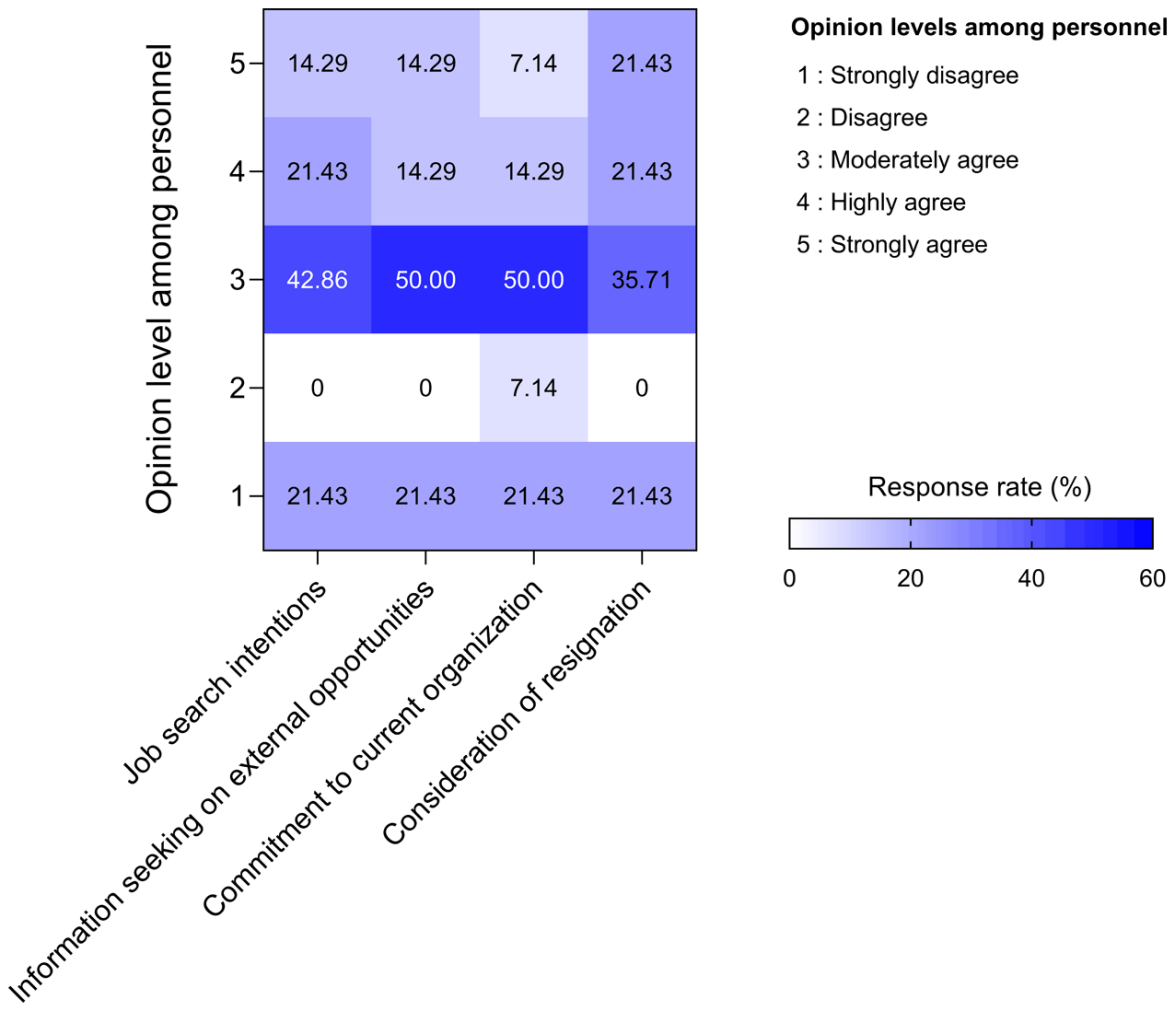
Heatmap illustrating the distribution of nuclear medicine personnel’s responses concerning their intention to continue employment within their current organization. The horizontal axis categorizes four key domains: job search intentions, information seeking on external opportunities, commitment to the current organization, and consideration of resignation. The vertical axis indicates the level of agreement, ranging from 1 (strongly disagree) to 5 (strongly agree). Color intensity represents the proportion of respondents selecting each response category, with darker shades of blue denoting higher response frequencies.

The findings presented in the heatmap illustrate key trends regarding the retention intentions of nuclear medicine personnel. Response patterns revealed that the majority of participants clustered around moderate agreement across all four assessed dimensions: job search intentions, information-seeking on external opportunities, commitment to the current organization, and consideration of resignation (Figure 3). This distribution suggests a prevailing sense of ambivalence within the workforce, with personnel neither expressing strong intentions to remain nor explicit plans to depart their current roles. A noteworthy proportion of respondents reported moderate to high levels of agreement concerning behaviors indicative of potential turnover, such as seeking new employment opportunities and contemplating resignation. These patterns highlight areas of organizational vulnerability, particularly in relation to workforce stability and retention. Such findings align with broader literature indicating that moderate turnover intention levels often reflect underlying dissatisfaction with factors such as work environment, compensation, career advancement opportunities, or organizational support.

In contrast, moderate levels of agreement were also observed in responses related to organizational commitment, suggesting that while staff are not disengaged, their allegiance may be contingent upon future improvements in workplace conditions. Highlighting the necessity for targeted organizational strategies that prioritize work place environmental protection, job satisfaction, professional development, and workload management to enhance retention. Strengthening these domains may serve to bolster institutional resilience by reducing turnover propensity and promoting a stable, experienced workforce within the highly specialized field of nuclear medicine.

### 3.5 Job satisfaction and the perceived value of work among nuclear medicine personnel

**Figure 4:**
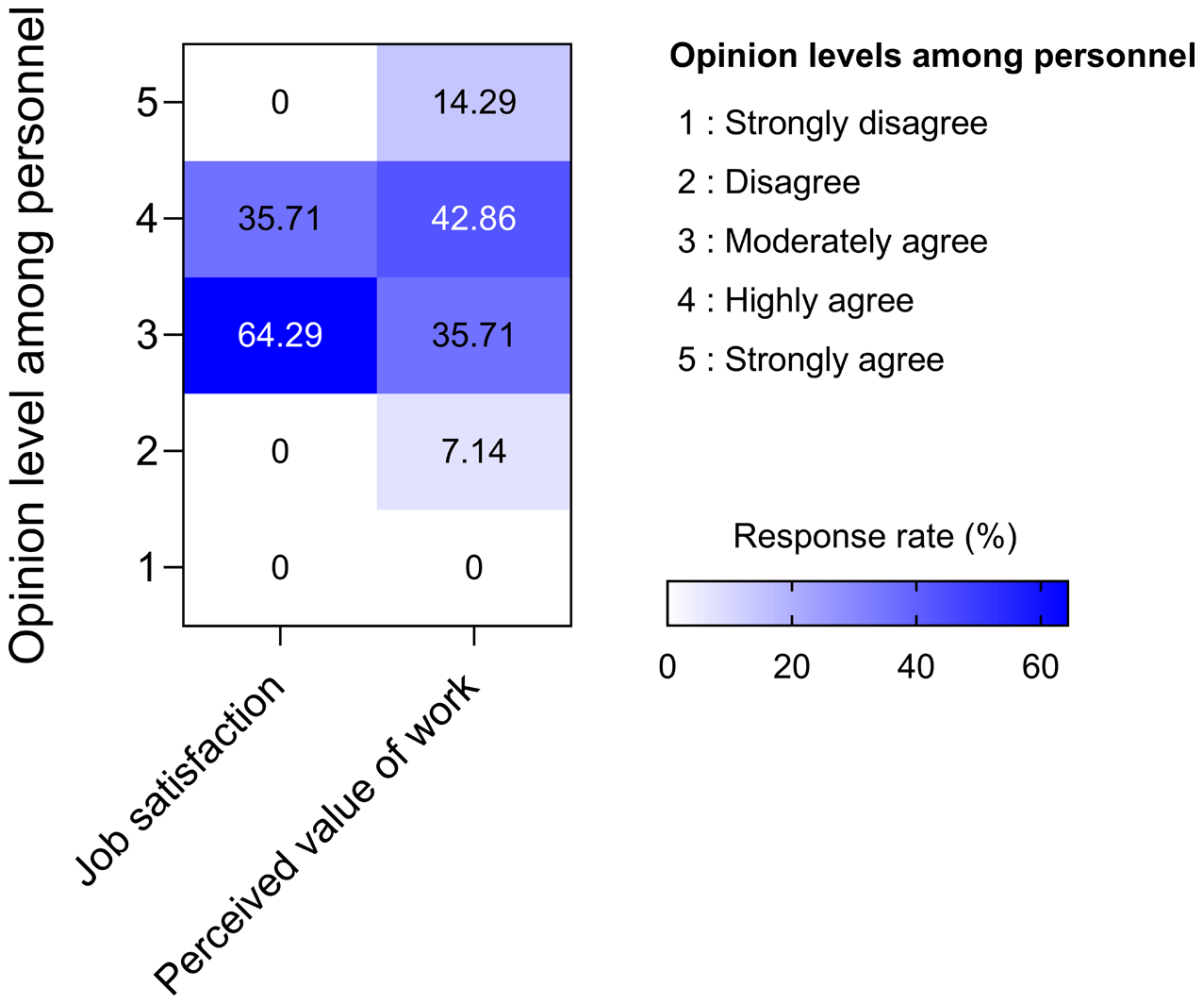
Heatmap illustrates the frequency distribution of nuclear medicine personnel’s responses regarding job satisfaction and perceived value of work. The vertical axis represents levels of agreement on a Likert scale ranging from 1 (strongly disagree) to 5 (strongly agree). The horizontal axis identifies the two measured constructs: job satisfaction and perceived value of work. Color intensity corresponds to the proportion of responses within each category, with darker shades indicating higher response rate.

Figure 4 illustrates the response of the participants to their perception of their job satisfaction and perceived value of work. The job satisfaction results indicate 64.29% moderately agreed, and a further 35.71% highly agreed. Which suggests a largely positive perception of their nuclear medicine occupational roles despite the challenges of working in a high-risk working environment. Furthermore, these results align with previous studies in specialized healthcare sectors that indicate the intrinsic motivation and professional commitment often remain strong (31–33). In contrast, the results of the participants’ acuities of their value of work unveiled across the levels of agreement a much broader range of responses. This disparity in the range of perceptions possibly suggests there are underlying differences in institutional support, recognition practices, or personal expectations regarding the societal and clinical contributions of nuclear medicine. Additionally, the relatively lower proportion of responses at the highest level of agreement regarding perceived work value emphasizes the urgent need for greater management engagement and organizational communications to reinforce the meaningfulness and broader impact of this specialized field.

Collectively, these results suggest that while job satisfaction remains relatively stable, perceptions of professional value are more variable and significantly influenced by organizational culture, recognition practices, and individual role alignment. The role of recognition practices in this process cannot be overstated. Efforts to strengthen these perceptions through enhanced feedback mechanisms, recognition programs, and professional development opportunities may help sustain workforce motivation and retention in nuclear medicine centers (34, 35).

### 3.6 Work burnout assessment among nuclear medicine personnel

**Figure 5:**
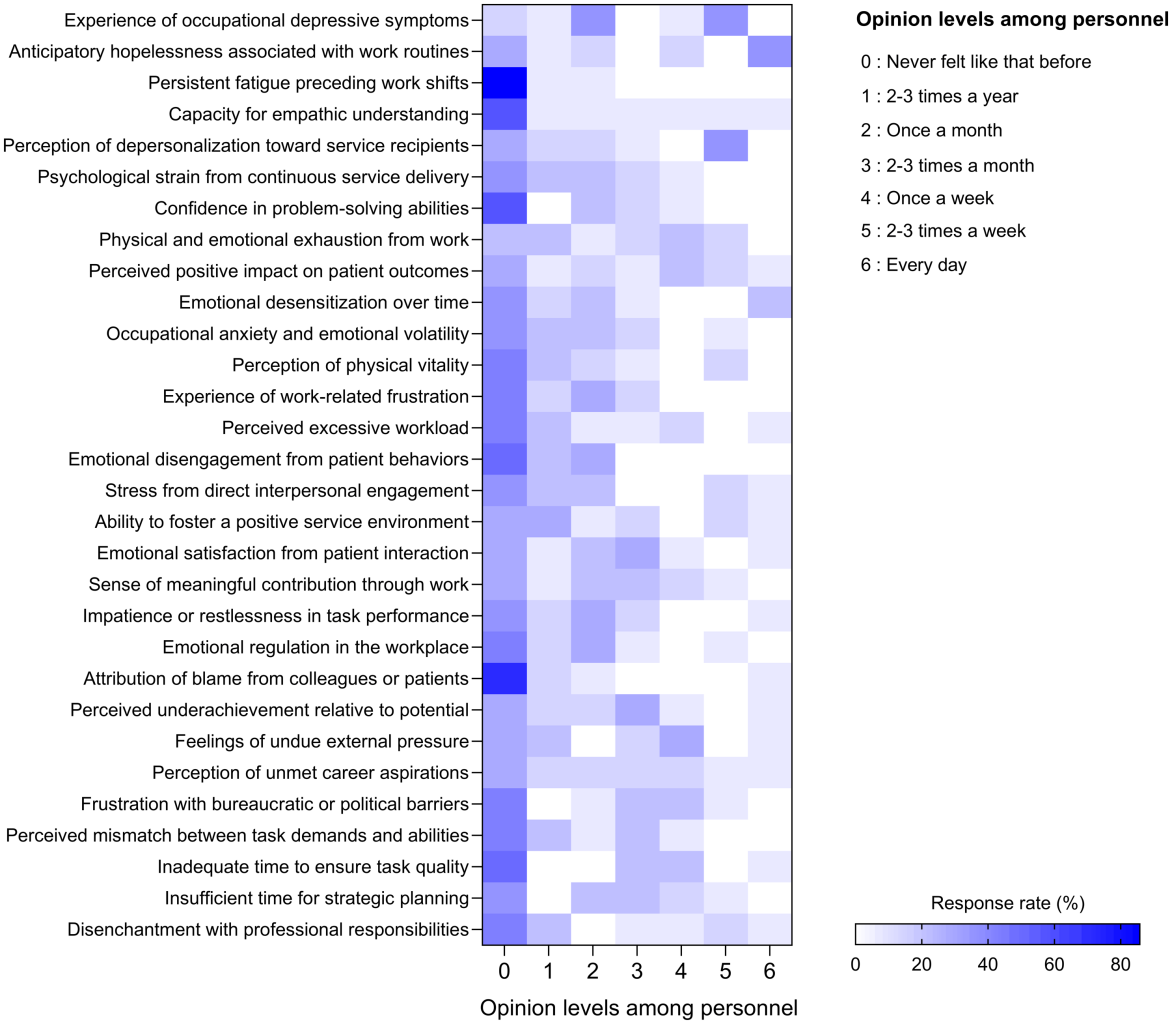
Heatmap represents the frequency distribution of occupational stress-related experiences among nuclear medicine personnel. The vertical axis presents 30 indicators reflecting emotional, cognitive, and physical responses to work-related psychosocial demands. The horizontal axis denotes the frequency of each experience, ranging from 0 (never felt like that before) to 6 (every day). Color intensity corresponds to the proportion of respondents endorsing each frequency level, with darker shades of blue indicating higher response rates (*n* = 20).

Figure 5 presents the frequency distribution of the occupational stress-related experiences among nuclear medicine personnel across graded response categories (0–6 scale). The most frequently reported indicators included persistent fatigue preceding work shifts, psychological strain from continuous service delivery, and emotional desensitization over time. These items show concentration within mid-range categories (levels 3–5), with responses clustering predominantly at monthly-to-weekly frequencies and limited polarization at the daily level (level 6). These patterns are consistent with features of emotional exhaustion, a core component of occupational burnout, and may reflect sustained exposure to high-intensity clinical environments. Additionally, medical staff concerns about radiation exposure may contribute to perceived stress, particularly when combined with workflow inefficiencies and high cognitive demands (36, 37). A notable proportion of respondents also endorsed items such as experience of occupational depressive symptoms and anticipatory hopelessness associated with work routines, with responses distributed across multiple frequency categories rather than concentrated at extreme endpoints. Concurrently, frequent responses to items, including perceived excessive workload, inadequate time for task completion, and frustration with bureaucratic or political barriers demonstrate dispersion across levels 2–4, indicating recurring systemic pressures rather than isolated distress. These psychosocial and procedural burdens are therefore suggestive of moderate but sustained occupational strain. Despite these challenges, some personnel consistently reported emotional satisfaction from patient interaction, a sense of meaningful contribution, and a strong capacity for empathic engagement, with these protective indicators skewed toward lower-to-mid response levels (levels 1–3). These protective factors, though less prevalent, suggest opportunities for resilience-building and role enrichment when appropriately supported.

In summary, the overall response distribution is characterized by central clustering within moderate frequency categories and limited endpoint polarization, consistent with sustained but non-severe occupational strain. Key recommendations include optimizing staffing and task distribution, promoting access to mental health resources, and reinforcing leadership accountability in fostering a psychologically supportive workplace. Such systemic strategies may help balance the physiological and emotional demands of a newly established cyclotron and molecular imaging nuclear medicine center with the long-term sustainability and satisfaction of its workforce.

### 3.7 Qualitative study on the perspectives and training needs of nuclear medicine personnel

#### 3.7.1 Qualitative study on the perspectives and training needs

Table 2 establishes the participants’ thematic reflections yielded significant insights into the strengths and ongoing challenges related to radiation safety training. Staff considered the training courses effective in enhancing their comprehension of radiation hazards and safety protocols. The acquired knowledge resulted in heightened confidence in routine tasks, especially in requesting suitable shielding and effectively using radiation monitoring equipment. These findings highlight the significant impact of structured education in promoting the adoption of evidence-based safety practices within clinical environments. Nonetheless, various practical limitations surfaced as considerable obstacles. Participants often indicated inadequate availability of essential personal protective equipment (PPE) and real-time dosimeters, especially during times of elevated clinical demand. Time constraints and heavy workloads hindered the consistent application of safety protocols. The findings indicate that enhanced knowledge and favorable attitudes may not suffice to ensure adherence to safety standards in the absence of sufficient institutional support. Resolving these issues necessitates investment in safety-related resources and modifications to workflows that emphasize protective practices in a high-pressure clinical setting. Additionally, the influence of organizational culture on safety behaviors is significant. Although leadership frequently highlighted the significance of radiation safety, participants observed that this messaging was not consistently reinforced by observable, ongoing actions. Conversely, work units with robust peer collaboration and mutual accountability exhibited a greater likelihood of consistently adhering to safety protocols. The findings emphasize the significant impact of team dynamics and the necessity for leadership that effectively communicates and exemplifies priorities. Participants provided recommendations for improving training effectiveness, such as implementing regular refresher courses and utilizing simulation-based learning with actual workplace equipment (38, 39). Reliable access to radiation monitoring and protective devices is essential for maintaining safe clinical practices.

**Table 2:** Summary of thematic analysis on the perspectives and training needs of nuclear medicine personnel in a newly established cyclotron and molecular imaging nuclear medicine center (*n* = 20).

Theme	Sub-theme	Key findings	Example quotes	Frequency	
				*N*	%
Perceived benefits	Knowledge enhancement	Training improved participants’ understanding of radiation hazards and safety measures.	“Now I know the importance of monitoring exposure regularly.” “I’ve realized how crucial it is to consistently monitor exposure levels.”	16	80
	Confidence in practice	Participants felt more confident in applying safety protocols during their daily tasks.	“I am no longer hesitant to ask for shielding.” “I now feel confident in requesting additional shielding.”	12	60
Barriers to implementation	Resource constraints	Lack of sufficient PPE and monitoring devices was a recurring issue.	“Sometimes we run out of shielding, especially during busy hours.” “There are times when shielding are unavailable, particularly during peak hours.”	6	30
	Time pressures	High workloads made it challenging to consistently follow safety protocols.	“I often skip steps when I’m in a rush to finish procedures.” “I sometimes miss steps when trying to finish procedures quickly.”	5	25
Cultural and organizational factors	Leadership support	Perception of inadequate encouragement from supervisors to prioritize radiation safety.	“Management talks about safety, but I rarely see them enforce it.” “Safety is a common topic in management discussions, but I rarely witness them putting it into practice.”	5	25
	Team dynamics	Positive team collaboration facilitated better adherence to safety protocols.	“When everyone on the team takes safety seriously, it motivates us all to follow through.” “When everyone prioritizes safety, it encourages us all to do the same.”	13	65
Recommendations for improvement	Practical simulations	Participants requested more hands-on practice during training sessions.	“It would be helpful to practice with the actual equipment we use daily.” “Training with the equipment we use daily would be really useful.”	8	40
	Regular refreshers	A desire for periodic refresher courses to keep safety knowledge current and relevant.	“A short refresher course every year would be very helpful.” “Having a short refresher course twice a year would be very useful.”	20	100
	Resource availability	Ensure consistent availability of PPE and real-time dosimeters in the center.	“We need more real-time dosimeters, so everyone has access to one at all times.” “We need to increase the number of real-time dosimeters to ensure they are always accessible to everyone.”	12	60

These findings highlight that radiation safety is not solely attained through training; rather, it requires a comprehensive, system-level approach where education, leadership, resource availability, and team engagement function synergistically. To cultivate a resilient safety culture, institutions must transcend minimal compliance and integrate safety as a collective organizational value, consistently reinforced across all operational levels.

#### 3.7.2 Strategic interventions to enhance QWL in high-stress healthcare settings

To improve QWL necessitates an approach that is well structured and systematic method applying proven management tools. These tools not only help identify problems, prioritize interventions, and monitor progress, but also empower the organization to take control of the situation. Some of the key management tools commonly used to enhance QWL applied in nuclear medicine organizational settings are as follows: (i) SWOT analysis (strengths, weaknesses, opportunities, threats), to identify those factors affecting QWL, enabling a strategic overview to prioritize actions that will enhance the work environment. A study by Bhavaraju et al., conducted a SWOT analysis of aspects pertaining to the work-life balance of medical professionals (40). (ii) Engagement surveys/QWL surveys, to ascertain employee perceptions of their work life, and pinpoint specific areas (workload, relationships, recognition, growth) needing attention, and empowering employee feedback to inform evidence-based improvements. A study revealed a significant association between the dimensions of QWL and job engagement (41). (iii) Kaizen (continuous improvement) aimed at fostering a culture of ongoing, incremental improvement through employee involvement. It engages staff in problem-solving, enhances ownership, and improves work processes, reducing frustration and inefficiencies. A study of organizations reported that there was a substantial association between continuous improvement and work life quality (42). (iv) 360-degree feedback to collect performance and behavioral feedback from peers, subordinates, and supervisors to encourage enhanced leadership performance, strengthen team dynamics, and enhance mutual understanding. Ugwoke et al. found that QWL and psychological empowerment markedly moderated the strong association between transformational leadership and performance of faculty staff (43). (v) Quality circles use a small group of medical staff gathering weekly for an hour to identify, analyze, and resolve work-related issues, which are then passed to management for implementation. A positive relationship was shown between quality circles engagement and alterations in QWL opinions in staff directly engaged in QC activities (44). (vi) Transformational leadership to improves QWL by encouraging intrinsic motivation, personal growth, and emotional well-being through caring about each person, stimulating their mind, and giving them power. It also creates a supportive, moral workplace where workers feel valued and involved, resulting in improved job satisfaction and less burnout (45, 46). Furthermore, AbdELhay et al. reported that transformational leadership was a significant factor in staff retention (47).

### 3.8 Integration of qualitative insights and quantitative dose assessment

#### 3.8.1 Environmental radiation exposure in ^18^F-FDG workplace

**Figure 6:**
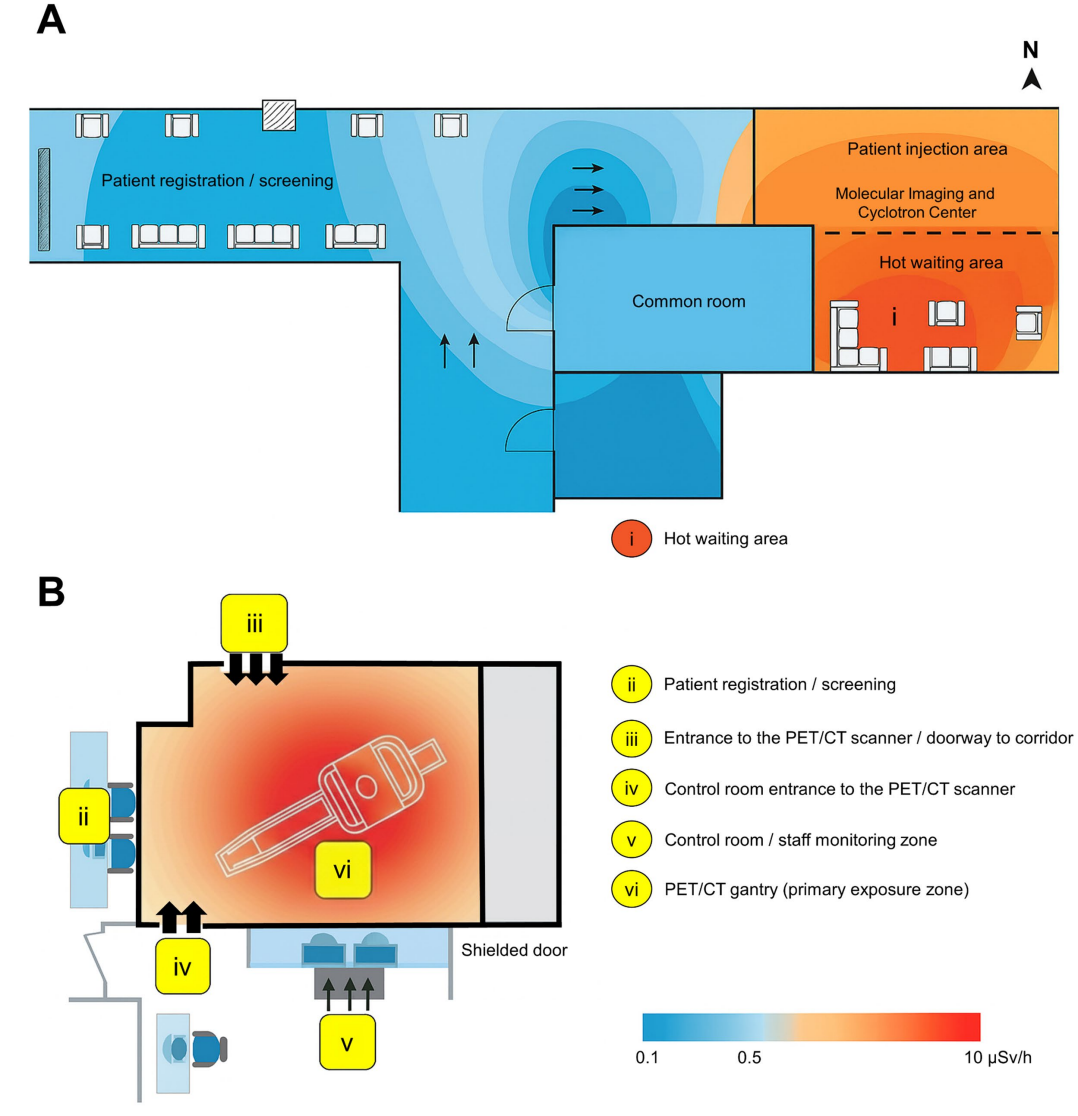
Environmental radiation exposure in ^18^F-FDG workplace. (**A**) Floor plan of the nuclear medicine department showing ambient dose rate distribution (μSv/h) measured during routine ^18^F-FDG procedures; zone (i) hot waiting area adjacent to the molecular imaging and cyclotron center. (**B**) PET/CT room layout indicating functional staff positions and radiation exposure zones: (ii) patient registration/screening, (iii) entrance to the PET/CT scanner, (iv) control room entrance, (v) control/monitoring zone, and (vi) PET/CT gantry (primary exposure zone). The color gradient bar represents the ambient dose intensity from 0.1 μSv/h (blue) to 10 μSv/h (red).

Figures 6A,B illustrates the spatial distribution of ambient dose rates within the PET/CT facility. Distinct dose gradients were observed, ranging from 0.1 μSv/h in peripheral areas (patient registration, control room) to 10 μSv/h in high-activity zones near the PET/CT gantry and hot waiting area. The highest dose concentrations correspond to areas where radiopharmaceutical administration, patient positioning, and scanning occur, emphasizing the need for optimized workflow design and shielding placement. Conversely, the common and administrative rooms maintained minimal exposure levels, demonstrating the effectiveness of existing architectural barriers and lead-shielded doors.

Figure 6B further delineates the functional zones of staff exposure. The primary exposure region (zone vi)—around the PET/CT gantry—recorded the most significant dose levels, followed by the entrance corridor (zone iii), where transient exposure occurs during patient movement. The control and monitoring area (zone v) maintained background-level doses, indicating adequate radiation protection. These findings validate that structural shielding and procedural zoning effectively mitigate stray radiation but emphasize the importance of continuous monitoring during high-demand periods. Integrating environmental mapping with qualitative insights revealed a comprehensive understanding of occupational exposure and safety culture in nuclear medicine practice. Participants in the qualitative study expressed strong theoretical understanding of radiation risks and confidence in routine procedures following structured training. However, they also highlighted resource limitations, inconsistent PPE availability, and time pressures, which may compromise safety adherence in high-dose areas—such as those identified in Figure 6B. The overlap between quantitative dose gradients and perceived workplace challenges suggests that radiological safety extends beyond engineering controls. While shielding and layout effectively reduce exposure, behavioral compliance, leadership engagement, and workflow management are critical in sustaining low-dose occupational environments. Enhancing real-time monitoring and simulation-based training can bridge this gap, reinforcing spatial awareness of high-risk zones. Overall, the integration of dose mapping with staff perceptions emphasizes that radiation protection is a multidimensional process combining physical controls with organizational, educational, and cultural reinforcement. The results advocate for a system-level approach where continuous training, accessible dosimetry, and proactive leadership align to maintain exposure within ALARA limits and strengthen the long-term resilience of safety culture in nuclear medicine workplaces.

#### 3.8.2 Occupational exposure assessment of healthcare personnel during ^18^F-FDG procedures

**Figure 7:**
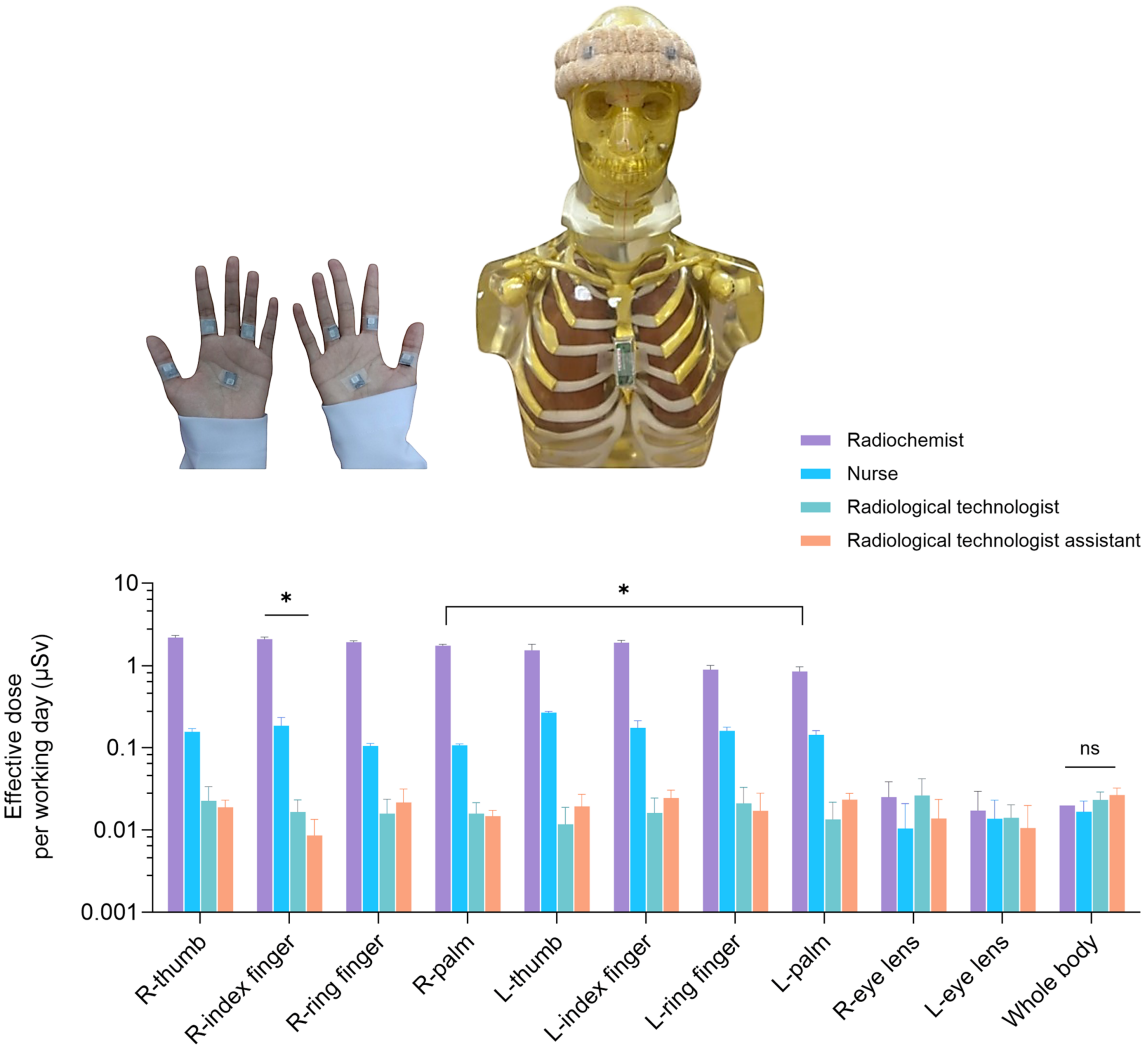
Effective dose per working day (μSv) measured at different body parts among staff involved in ^18^F-FDG procedures. The dose distribution was compared among four occupational groups: radiochemists, nurses, radiological technologists, and radiological technologist assistants. The radiochemists exhibited significantly higher extremity doses (R-hand and L-hand) compared with other groups (*p* < 0.05), while no significant difference (ns) was observed in whole-body doses. Error bars represent standard errors of the mean (SEM).

From the qualitative results, it became evident that nuclear medicine personnel possessed a strong conceptual understanding of radiation safety but faced practical barriers—limited protective equipment, real-time dosimeters, and time constraints often impeding consistent safety compliance (Table 2). Consequently, these findings motivated a further quantitative assessment of the effective dose distribution across different body parts to objectively evaluate whether the perceived safety practices translated into adequate radiological protection. As can be seen in Figure 7, distinct variations in occupational dose were observed among staff categories. Radiochemists demonstrated the highest extremity doses, particularly at the right thumb, index finger, and palm (*p* < 0.05), consistent with their direct handling of unshielded ^18^F-FDG during synthesis and dispensing. Nurses exhibited moderate hand exposure levels due to their proximity during injection and patient preparation, while radiological technologists and assistants showed minimal extremity doses (< 0.05 μSv per working day), reflecting effective distance and shielding practices. Additionally, no statistically significant differences were found in eye-lens or whole-body doses across groups (*p* > 0.05), indicating that existing shielding and workflow protocols effectively minimize deep-dose exposure. The combined qualitative and quantitative findings suggest that, although training improved radiation-safety awareness, practical workflow limitations and manual handling tasks remain the main determinants of exposure, particularly for radiochemists and nurses (48–50).

#### 3.8.3 Association between radiation dose and psychosocial outcomes

Associations between radiation dose metrics and psychosocial outcomes were examined using both unadjusted and group-adjusted models. In unadjusted analyses (Supplementary Figures S1–S5), extremity dose demonstrated a moderate inverse association with QWL composite score and job satisfaction. Whole-body dose was not significantly associated with QWL. To account for occupational clustering, group-adjusted linear regression models were performed (Table 3). After adjustment for occupational group, extremity dose was not independently associated with QWL or job satisfaction. Whole-body dose was similarly not associated with QWL. Distribution-robust analyses yielded consistent findings (Table 4). In rank-based ANCOVA adjusted for occupational group, extremity dose (*β* = −0.15, *p* = 0.741) and whole-body dose (*β* = 0.15, *p* = 0.828) were not significantly associated with QWL. Robust linear regression models with HC3 heteroscedasticity-consistent standard errors produced comparable non-significant estimates. Collectively, after adjustment for occupational role, radiation dose metrics were not independently associated with psychosocial outcomes in this cohort.

**Table 3:** Exploratory linear regression models examining associations between radiation dose metrics and psychosocial outcomes adjusted for occupational group.

Model	Dose predictor	*β*	95% CI	*p*-value
QWL ~ Extremity Dose + Occupational Group	Extremity dose	0.74	−2.95 to 4.42	0.695
QWL ~ Whole-body Dose + Occupational Group	Whole-body dose	−0.17	−215.09 to 214.75	0.999
Job Satisfaction ~ Extremity Dose + Occupational Group	Extremity dose	−0.04	−0.65 to 0.56	0.884

Unstandardized regression coefficients (*β*), 95% confidence intervals (CI), and *p*-values are presented. QWL models were estimated using robust linear regression with HC3 heteroscedasticity-consistent standard errors. Job satisfaction was analyzed using group-adjusted linear modeling for comparability across outcomes. All models are interpreted as exploratory given the pilot sample size (*n* = 20).

**Table 4:** Distribution-robust analyses evaluating associations between radiation dose metrics and QWL after adjustment for occupational group.

Model	Predictor	*β*	95% CI	*p*-value
Rank-based ANCOVA†	Extremity dose	−0.15 (rank scale)	—	0.741
Rank-based ANCOVA†	Whole-body dose	0.15 (rank scale)	—	0.828
Robust linear regression‡	Extremity dose	0.74	−2.95 to 4.42	0.695
Robust linear regression‡	Whole-body dose	−0.17	−215.09 to 214.75	0.999

†Rank-based ANCOVA performed using rank-transformed dependent and predictor variables to provide distribution-robust adjustment for occupational clustering. ‡Linear regression fitted with HC3 heteroscedasticity-consistent robust standard errors. Exploratory analysis in pilot sample (*n* = 20).

## 4 Discussion

This research offers significant insights into how environmental safety and safety training affect the QWL and job satisfaction of healthcare professionals in a newly established cyclotron and molecular imaging nuclear medicine center (<5 years). The results indicate a multifaceted interaction of elements affecting employee happiness, retention, and general well-being. To further contextualize these findings, it is important to consider workforce demographics in this field. The American Medical Association reported that radiology remains male-dominated, with males constituting 73.2% of the resident workforce (51, 52). Women in nuclear medicine constitute around 20–30% of the worldwide workforce. However, in Thailand, the percentage of women employed in radiology departments surpasses 70% (53), and in our nuclear medicine facility in Thailand, females represent 85.71% of the workforce. In this study, the predominantly female and highly educated participants exemplify a typical demographic within the healthcare profession across various settings in Thailand. However, at our nuclear medicine facility the high prevalence of inexperienced staff with 1–5 years’ experience (71.43%) suggests a dynamic workplace that can potentially present challenges and opportunities. These include developing emotional resilience, a crucial skill in the healthcare profession, time management and efficiency, gaining technical skills, fostering lifelong learning, adaptability, and protocol compliance. Therefore, consideration should be given to enable staff to have flexible work arrangements wherever possible, including remote work, adaptable hours, and part-time positions, to enhance work-life balance for female workers (54). In fact, Zheng et al. found that employees who employed work-life balance strategies demonstrated enhanced health and well-being relative to those who did not, and were more proficient in achieving work-life balance (55). In contrast, Hartanto et al. stated that there was a heightened cardiovascular risk correlated to workplace tension (56). Therefore, this study suggests that human resources strategies are an essential element for facilitating women’s career-life balance to attain an optimal work-life balance. This study highlights the need for a proactive strategy to understand and address workforce requirements, particularly for women. Findings from Muda et al. indicate that female employees often encounter unique challenges in balancing professional and personal responsibilities due to traditional gender roles and societal expectations, which can impact their job satisfaction and career progression (57).

Several studies have found a lack of knowledge about radiation safety. For example, a recent study of 384 Egyptian healthcare workers found that most healthcare personnel lacked a comprehensive understanding of radiation exposure safety. Furthermore, the majority of healthcare personnel demonstrated weak compliance with radiation safety practices (48, 58). Another study of 413 healthcare professionals found that 64.4% demonstrated a lack of knowledge regarding radiation safety (59). Similarly, of concern the results of our study found that initially 14.29% of staff adhered to radiation protocols. However, as a result of subsequent radiation safety training, staff awareness increased to 80% (Table 2), and adherence to protocols also dramatically increased to 87%. Notably, this study highlights the need for consistent training and emphasizes possible areas for improvement. This aligns with the results of Rink et al., who recognized ongoing education as an essential element in alleviating pressures among healthcare professionals (6). The response to 3.4 of 5 scored a low mean of 3.14, indicating that staff are concerned about insufficient shielding and safeguards.

As a result of this study, a comprehensive survey of environmental radiation levels in the center verified that all areas were within safety limits (30). Additionally, evaluating the effective radiation doses for all staff involved with bone scintigraphy scans highlighted those most at risk (Figure 7). Consequently, all staff will be provided with extra personal radiation safety shielding, and all working practices will be assessed to improve workflow and mitigate personal effective radiation dose. Moreover, this qualitative study demonstrates the effectiveness of training programs in improving radiation safety awareness and boosting staff confidence while also highlighting any systemic barriers, such as resource shortages and time constraints. Regular training programs improve radiation safety awareness and confidence by strengthening staff knowledge and skills (48, 60, 61). Additionally, training highlights systemic barriers because informed and confident professionals are better equipped to recognize organizational limitations. Noteworthy, the results of this study and subsequent training have successfully advanced participants from passive compliance to critical, more safety-oriented practice. Addressing these challenges requires a multi-faceted approach that integrates regular training, adequate resource allocation, and strong leadership support to foster a safety culture. However, as stated by De Brún et al., it is good leadership, rather than hierarchical compliance, that enhances collaboration, safety, and knowledge, but must be continually maintained at all organizational levels (62). Moreover, these notions correspond with the more general research, which highlights the interaction of an individual capabilities, collaboration, and organizational elements in producing the ideal safety result (58, 63).

Furthermore, the varied outcomes regarding intentions to remain employed at the nuclear medicine center illustrate the intricate dynamics of employee retention in the healthcare sector. The modest agreement, along with a long-term commitment and willingness to explore other job opportunities, indicates that employee satisfaction is incomplete while a foundational degree of loyalty exists. This scenario presents management with a problem, but more importantly, an opportunity to execute retention initiatives. Boone et al. suggest many approaches to improve employment retention in healthcare. Integrating several interventions, including personal and professional assistance, educational opportunities, financial incentives, and regulatory frameworks, is crucial since there is no one-size-fits-all approach (64). Additionally, Chegini et al. found that turnover intention is common in most organizations and adversely affects personnel. Since low wages and salaries are the most significant factors in poor QWL, healthcare management should implement financial and non-financial incentives to inspire personnel and improve QWL (65). The modest levels of work satisfaction in many areas correspond with prior research in healthcare environments. The results align with those of Rostami et al., who identified a significant association between work-related quality of life and job satisfaction among healthcare professionals (9). In addition, a study by Desai et al. noted that dissatisfaction with levels of remuneration is consistent with broader trends in healthcare, emphasizing the critical role that remuneration has in determining quality of life at work and job satisfaction (66). Moreover, as reported by the World Health Organization (WHO), higher remuneration and better working conditions are some of the key factors driving significant numbers of healthcare workers’ migration from their home countries to work abroad (67). Correspondingly, emotional demands, quantitative demands, predictability, work pace, vertical trust, and the quality of leadership all influence the QWL for adult critical care unit staff (68). Likewise, several studies have shown that those people who are more psychologically resilient tend to be more engaged in their work. Because psychological resilience enhances work engagement by helping individuals conserve energy, maintain enhanced motivation and focus, and manage stress effectively, enabling them to remain vigorous, dedicated, and immersed in their work even under demanding conditions. Additionally, psychological resilience can positively predict increasing work engagement (69, 70). By addressing any management practices that are unfair, fostering a more favorable regulatory climate, and investing in employee resilience can contribute to a thriving work environment for individuals and organizations (71–73). According to Amoadu et al. (10), the elevated rating for democracy and justice in the workplace is a positive outcome consistent with the importance of a psychologically safe atmosphere (10). Moreover, psychological safety is crucial and shown to enhance work satisfaction, while also providing a feeling of reassurance and security to healthcare professionals (74).

The modest level of satisfaction with work–life balance observed among participants suggests that targeted interventions may be warranted to enhance overall well-being and reduce the risk of burnout. However, it was revealed that 28% of participants regularly experienced a sense of overwhelm (Figure 4). This discovery is especially pertinent considering the likelihood of work-related stress and burnout in healthcare environments, as emphasized by Rania et al. in their research on mental health and quality of life of healthcare professionals after COVID-19 (5). However, the lack of agreement on the cause of burnout has resulted in a profusion of definitions and metrics, making it impossible to accurately estimate its frequency and prevalence. Consequently, the Network on the Coordination and Harmonization of European Occupational Cohorts (OMEGA-NET) has established a consensus regarding the definition of occupational burnout as follows: “Occupational burnout in a worker is a state of physical and emotional exhaustion resulting from extended exposure to work-related issues.” A study by Salyers et al. indicates that the paramount factor in mitigating burnout is emotional exhaustion resulting from persistent stress and overwhelming workloads (75). Furthermore, a review of work environments and burnout by Aronsson et al. discovered substantial evidence of a link between job control and less emotional exhaustion and a link between inadequate workplace support and greater emotional exhaustion (76). The present research findings suggest that initiatives to strengthen work-life balance can enhance job satisfaction and reduce burnout. Practical measures include flexible scheduling, compressed workweeks, mental health days, shorter meetings, limits on after-hours communication, and opportunities for regular breaks. Wellness programs, childcare assistance, and paid family leave address personal responsibilities, while on-site health facilities and financial wellness programs promote overall well-being. Clear workload expectations, defined boundaries, and results-focused performance evaluations help minimize stress. In addition, part-time options and team-building activities support balance and foster a positive workplace culture. As employees are an organization’s most valuable asset, neglecting their well-being risks reduced productivity, lower quality, and organizational decline (77, 78).

This study evaluated associations between radiation dose metrics and psychosocial indicators of workforce well-being. Descriptive analyses showed variability across QWL domains, particularly in workload-related dimensions. In unadjusted analyses, extremity dose was inversely associated with QWL and job satisfaction. However, after adjustment for occupational group, these associations were no longer statistically evident in rank-based ANCOVA and group-adjusted robust regression models (Tables 3–4). Dose coefficients were small and accompanied by wide confidence intervals. Both extremity dose and QWL were stratified by occupational role, suggesting that between-group structure largely explained the unadjusted correlations. Adjusted analyses did not support an independent association between radiation dose metrics and psychosocial outcomes within occupational strata.

According to a Health Productivity and Performance Survey Committee survey, a resounding 90% of businesses believe that wellness promotion significantly affects employee performance and productivity (79). The State of Work-Life Wellness 2024 research further emphasizes the high value businesses place on wellness, indicating that 93% of US employees consider their workplace wellness as significant as their compensation (80). Employers recognize that content and healthy workers exhibit superior performance and productivity. Since people are a company’s most important asset, investing resources in promoting wellness and health yields significant returns. Many organizations promote healthier and happier workers via employee wellness programs. The National Wellness Institute defines well-being as emotional, occupational, physical, social, intellectual, and spiritual (81). The James Cancer Hospital, Ohio State University, implemented the Stanford Professional Fulfillment Index to survey physicians’ well-being (82). As stated by the Stanford model, Personal Resilience and an organization’s commitment to a culture of wellness and efficiency of practice generate employee well-being in the workplace.

## 5 Conclusion

This study provides preliminary evidence suggesting that effective management of occupational radiation exposure in PET/CT practice may benefit from the integration of environmental safety, radiation monitoring, and workforce well-being, particularly in a newly established cyclotron and molecular imaging nuclear medicine center. Although overall job satisfaction among healthcare professionals at the center of Songklanagarind Hospital was generally satisfactory, several critical management issues were identified during the early operational phase. Notably, radiochemists experienced the highest extremity doses, which may indicate the need for targeted workflow controls, improved shielding strategies, and task-specific extremity dosimetry to mitigate exposure risks associated with radiopharmaceutical preparation and dispensing. The findings suggest that job satisfaction in nuclear medicine practice may be influenced not only by fair remuneration, career development opportunities, and work–life balance, but also by the effectiveness of radiation protection infrastructure, monitoring systems, and safety governance. Importantly, structured radiation safety training was associated with improvements in staff awareness and compliance, increasing adherence to radiation protection protocols from 14.29 to 87%. Additionally, the results indicate that extremity dose may represent an important component of occupational exposure in PET radiopharmaceutical workflows and may not always be fully reflected by whole-body effective dose measurements alone. Consequently, comprehensive radiation protection programs in PET/CT centers may consider incorporating environmental radiation detection, task-specific personal and extremity dosimetry, and organizational controls to ensure exposures remain within ALARA limits.

Overall, this pilot study offers context-specific insights for the planning and management of newly established nuclear medicine centers. Implementation of system-level radiation protection strategies aligned with international standards, alongside attention to workforce well-being, may contribute to reducing occupational risk and supporting staff engagement and retention. Future multi-center studies with adequate statistical power are needed to validate these findings and enable robust multivariable modeling. Longitudinal designs may further clarify temporal relationships between radiation exposure, organizational factors, and workforce well-being. Integrating dosimetric monitoring with structured workforce assessment may support proactive occupational health planning and sustainable service development in high-risk healthcare environments.

## Data Availability

The datasets utilized in the present study can be obtained from the corresponding author upon making a reasonable request.
