# Supplementary material for: Occupational radiation safety and sustainable workforce management in PET/CT practice: a pilot study of healthcare professionals in a newly established cyclotron-based nuclear medicine center
Source: Front Public Health. 2026 Mar 27;14:1782228. doi: 10.3389/fpubh.2026.1782228 (PMC13066160; doi:10.3389/fpubh.2026.1782228)
Supplement: Supplementary file 1 [file Data_sheet_1.pdf]

Supplementary Material

Occupational Radiation Safety and Sustainable Workforce Management in PET/CT Practice: A Pilot Study of Healthcare Professionals in a Newly Established Cyclotron-Based Nuclear Medicine Center

Suphalak Khamruang Marshall [1,*], Kunlagan Mewes [2] , Nadia Noomad [1] , Wanita Durawee [1] and Awatif Hayeeabdunromae [1]

[1]Department of Radiology, Faculty of Medicine, Prince of Songkla University, Songkhla 90110, Thailand

[2]Department of Business Administration, Faculty of Management Sciences, Prince of Songkla University, Songkhla 90110, Thailand

*** Correspondence:**
Suphalak Khamruang Marshall
suphalak.k@psu.ac.th

1 Supplementary Data

1.1 Radiation Safety Training Program Description

The original training sessions were delivered in Thai; the present document constitutes an English translation that maintains fidelity to the original instructional content and structure. Training was conducted onsite using a structured format comprising didactic lectures, facilitated workshops, and supervised practical demonstrations. The program formed part of the early operational safety governance framework of the cyclotron-based PET/CT nuclear medicine center.

1.1.1 Training Objectives

- Reinforce core radiation protection principles (Justification, Optimization, and ALARA).
- Improve extremity dose monitoring and shielding practices.
- Standardize radiopharmaceutical handling and workflow procedures.
- Strengthen contamination control and spill response readiness.
- Enhance compliance with regulatory and institutional safety standards.

1.1.2 Instructor Qualifications

Sessions were conducted by certified radiation safety personnel, including a licensed Medical Physicist and the departmental Radiation Safety Officer (RSO), both formally trained in radiation protection, dosimetry, and nuclear medicine safety management.

1.1.3 Participants

All four occupational groups within the department—radiochemists, nurses, radiological technologists, and radiological technologist assistants—were invited to participate. Attendance was formally documented as part of institutional safety governance records.

1.1.4 Integration into Safety Governance

The training program was integrated into routine departmental safety governance, including formal documentation within the quality assurance framework, periodic compliance audits, and alignment with continuous professional development requirements.

1.2 Training Structure and Delivery Format

Training was delivered onsite in three structured sessions (2 hours per session) using a blended format consisting of lecture-based instruction, interactive workshops, and practical demonstrations in the hot lab and injection areas. Core topics included:

1.2.1 Radiation Protection Principles and Occupational Dose Limits in Nuclear Medicine

Radiation protection in nuclear medicine is guided by internationally recognized principles established by the International Commission on Radiological Protection (ICRP) and national regulatory authorities. These principles are designed to protect patients, healthcare personnel, and the public from unnecessary exposure to ionizing radiation while maintaining clinical effectiveness. Workflow standardization and procedural rehearsal are emphasized to reduce unnecessary handling time and extremity exposure.

1.2.1.1 Fundamental Radiation Protection Principles

- Justification: All nuclear medicine procedures must be clinically justified. A diagnostic or therapeutic exposure should only be performed when the expected medical benefit outweighs potential radiation risks. This includes appropriate radiopharmaceutical selection, activity optimization, and avoidance of unnecessary repeat examinations.
- Optimization (ALARA Principle): Occupational exposures must be kept As Low As Reasonably Achievable (ALARA), taking into account economic and operational considerations. In nuclear medicine practice, optimization is achieved through:
    - Time: Minimizing duration of exposure during radiopharmaceutical preparation and administration.
    - Distance: Maximizing distance from radioactive sources (inverse square law).
    - Shielding: Using appropriate protective devices such as syringe shields, L-blocks, lead glass barriers, vial pigs, and tungsten shielding for high-energy radionuclides (e.g., ^{18}F).

1.2.1.2 Occupational Dose Limits (ICRP-Consistent Values)

Occupational exposure must not exceed regulatory dose limits. Dose limits serve as upper safety boundaries and do not replace optimization. For radiation workers in nuclear medicine:

- Effective dose (whole body): 20 mSv per year averaged over 5 years (not exceeding 50 mSv in any single year)
- Lens of the eye: 20 mSv per year
- Extremities (hands and feet): 500 mSv per year
- Skin: 500 mSv per year
- For comparison, the annual dose limit for the general public is 1 mSv.

1.2.1.3 Application in Nuclear Medicine Practice

- Extremity Exposure: Radiochemists and personnel involved in radiopharmaceutical preparation often receive the highest extremity doses due to direct handling of high-activity syringes. Proper extremity dosimeter placement (typically on the dominant hand) is essential for accurate monitoring.
- Whole-Body Monitoring: Personal dosimeters are worn at chest level and reviewed periodically. Dose trend monitoring is emphasized over isolated values to detect changes in exposure patterns.
- Shielding and Engineering Controls: Appropriate shielding selection depends on radionuclide energy. PET radionuclides (e.g., ^{18}F) require higher-density shielding due to 511 keV photon emission.

1.2.1.4 Safety Culture and Governance

Radiation protection is integrated into institutional safety governance through:

- Routine dosimetry review
- Investigation levels below regulatory limits
- Compliance audits
- Ongoing training and competency assessment
- Incident reporting and corrective action protocols

1.2.1.5 Key Training Message

Radiation protection in nuclear medicine is a continuous, practice-based responsibility rather than a theoretical requirement. Every procedure must be clinically justified, and all occupational exposures must be optimized in accordance with the ALARA principle. Personnel are expected to wear and correctly position whole-body and extremity dosimeters at all times when working in controlled areas, and to use appropriate shielding and remote handling tools to minimize exposure during radiopharmaceutical preparation and administration. Extremity exposure requires particular vigilance in PET/CT settings. Staff must routinely review personal dose reports and promptly report any unusual or elevated readings to the Radiation Safety Officer. A strong radiation safety culture depends on shared accountability, consistent procedural compliance, and proactive risk awareness across all occupational groups.

1.2.2 Controlled and Supervised Area Requirements

1.2.2.1 Definitions (ICRP / IAEA Framework)

Radiation workplaces are classified to ensure appropriate protection measures based on potential exposure levels.

- Controlled Area: A Controlled Area is a designated space where:
    - Specific radiation protection measures are required
    - Access is restricted to trained personnel
    - Occupational dose may approach investigation levels if not properly managed.
    - Contamination risk from unsealed sources exists
    - Examples in Nuclear Medicine: Hot laboratory (radiopharmaceutical preparation room), Radiopharmacy dispensing area, PET injection room, Radioactive waste storage room, Generator elution area
- Supervised Area: A Supervised Area is a space where:
    - Occupational exposure is unlikely to exceed regulatory limits
    - Conditions are periodically reviewed
    - No routine special protective measures are required
    - Contamination risk is low
    - Examples: Console room (if shielded), Adjacent patient waiting area, shielded control areas, Reporting room

1.2.2.2 Regulatory Basis

According to IAEA GSR Part 3 and ICRP Publication 103/137:

- Controlled areas must be clearly demarcated.
- Access must be limited to trained personnel.
- Radiation monitoring and contamination control must be implemented.
- Investigation levels should be established below regulatory dose limits.

1.2.2.3 Requirements for Controlled Areas

- Physical Controls
    - Clear radiation warning signage
    - Restricted access doors
    - Shielded workstations (L-blocks, lead glass, tungsten shielding)
    - Designated contamination monitoring station
    - Absorbent bench covering in hot lab
    - Separate radioactive waste storage
- Administrative Controls
    - Entry limited to authorized personnel
    - Use of personal dosimeters (whole-body and extremity)
    - SOP for radiopharmaceutical handling
    - Spill response protocol
    - Dose investigation levels
    - Routine compliance audits
- Monitoring Requirements

- Area radiation survey meters
- Contamination monitoring (GM counter / wipe testing)
- Personal dosimetry review
- Documentation of dose records

1.2.2.4 Requirements for Supervised Areas

- Periodic dose rate monitoring
- No routine contamination expected
- Signage indicating radiation area (if applicable)
- No requirement for extremity dosimeter unless entering controlled area

1.2.2.5 Key Training Message

Radiation safety begins with situational awareness. Personnel must understand the classification of the area in which they are working (controlled vs. supervised), as protection requirements differ accordingly. Entry into controlled areas requires strict adherence to access procedures, including verification of authorization, appropriate personal protective equipment, and correct placement of whole-body and extremity dosimeters prior to handling radioactive materials. Before exiting the hot lab or any controlled area, staff must perform contamination monitoring of hands, clothing, and footwear to prevent spread of radioactive material. Any abnormal dose rate readings, unexpected contamination, or unusual dosimetry results must be reported immediately to the Radiation Safety Officer. Consistent application of these practices reinforces ALARA principles, prevents secondary contamination, and sustains a strong radiation safety culture within nuclear medicine operations.

1.2.3 Extremity Dosimetry and Shielding Optimization Strategies

Extremity dose is typically the dominant occupational exposure component in nuclear medicine, particularly in PET/CT practice involving high-activity ^{18}F-labeled radiopharmaceutical handling. Because hands are positioned closest to unsealed sources, targeted monitoring and shielding optimization are essential to ensure compliance with dose limits and the ALARA principle.

1.2.3.1 Extremity Dosimetry

- Indications for Monitoring: Extremity dosimetry is required for personnel who:
  - Prepare or dispense radiopharmaceuticals
  - Perform generator elution
  - Handle high-activity PET syringes
  - Conduct manual dose drawing or injection
  - Radiochemists and technologists in hot lab environments typically receive the highest extremity doses.
- Extremity Dosimetry: Extremity dosimetry is required for personnel who:
  - Prepare or dispense radiopharmaceuticals
  - Perform generator elution
  - Handle high-activity PET syringes
  - Conduct manual dose drawing or injection
  - Radiochemists and technologists in hot lab environments typically receive the highest extremity doses.

- Regulatory Dose Limits (ICRP-Consistent)
    - Extremities (hands and feet): 500 mSv/year
    - Skin: 500 mSv/year
    - Although limits are high, internal investigation levels should be set substantially lower to trigger workflow review.
- Workflow Optimization to Reduce Extremity Dose
    - Pre-plan preparation steps
    - Minimize unshielded syringe time
    - Avoid air bubble removal outside shield
    - Keep fingers behind shield edge
    - Step back immediately after injection
    - Rotate high-intensity tasks among staff
    - Small time reductions during repeated tasks significantly reduce cumulative dose.
- Common Causes of Elevated Extremity Dose: Routine observational audits are recommended.
    - Removing syringe from tungsten shield
    - Leaning over L-block edge
    - Holding vial during activity measurement
    - Repeated manual drawing of high-activity doses
    - Inadequate shielding thickness for PET

1.2.3.2 Shielding Optimization Strategies (PET-Specific)

PET radionuclides emit 511 keV annihilation photons, requiring higher-density shielding compared to conventional gamma emitters.

- Shielding Materials: Tungsten provides greater attenuation per unit thickness and is preferred for handheld shielding.

Shielding Material	Density (g/cm³)	Typical Use in PET
Lead	11.34	L-block barriers, room shielding
Tungsten	19.3	Syringe shields, vial pigs

- Approximate PET Shielding Requirements (511 keV): Values are approximate and depend on geometry and scatter conditions.

Target Attenuation	Lead Thickness	Tungsten Thickness
~50% reduction	~6 mm	~3 mm
~90% reduction	~12 mm	~6 mm

1.2.3.3 Integration with ALARA and Workforce Sustainability

- Extremity dose is often a surrogate marker for:
    - Task concentration
    - Workflow inefficiency
    - Staffing imbalance
    - Insufficient engineering controls
- Optimization should therefore integrate:

  - Engineering improvements
  - Administrative controls
  - Staff rotation
  - Continuous safety training
  - Periodic competency reassessment

1.2.3.4 Key Training Message

Extremity dose in nuclear medicine is controllable. Effective dosimetry, appropriate tungsten shielding, workflow optimization, and proactive dose review are essential to maintain regulatory compliance and sustain long-term occupational safety in PET/CT practice.

1.2.4 Radiopharmaceutical Preparation and Administration Safety

Radiopharmaceutical preparation and administration represent the highest-risk occupational phases in nuclear medicine practice, particularly in PET/CT settings using high-activity unsealed sources. Safety in these stages requires integration of radiation protection principles, contamination control, aseptic technique, workflow standardization, and regulatory compliance.

1.2.4.1 Hot Lab (Radiopharmacy) Safety

- Controlled Environment Requirements: Radiopharmaceutical preparation must occur in a designated controlled area equipped with:
  - Shielded L-block with lead glass viewing panel
  - Tungsten vial pigs and syringe shields
  - Calibrated dose calibrator
  - Contamination monitoring equipment
  - Shielded radioactive waste container
  - Spill kit and absorbent bench covering
  - Access must be restricted to trained personnel.
- Workflow Standardization: To minimize exposure and errors:
  - Pre-plan preparation steps
  - Confirm prescribed activity and radionuclide
  - Verify patient identity prior to dispensing
  - Use pre-calibrated unit doses where possible
  - Avoid unnecessary manipulation
  - Preparation should follow a written Standard Operating Procedure (SOP)

1.2.4.2 Radiation Protection During Preparation

- Time–Distance–Shielding Application
  - Keep source within shield at all times
  - Minimize unshielded syringe handling
  - Maintain hands behind L-block edge
  - Use tungsten syringe shields (≥5–6 mm for PET)
  - Avoid leaning over radioactive source
- Activity Measurement
  - Use properly calibrated dose calibrator

- Confirm radionuclide setting
- Avoid direct hand contact with unshielded vial
- Record activity, time, and operator

1.2.4.3 Administration (Injection) Safety

- Pre-Injection Verification
  - Confirm patient identity (two identifiers)
  - Verify radiopharmaceutical and activity
  - Confirm administration route
  - Check pregnancy/breastfeeding status (where applicable)
  - Explain procedure to patient
- Injection Technique
  - Maintain syringe shielding until immediately before injection
  - Keep distance once injection is complete
  - Use extension tubing where appropriate
  - Avoid hand repositioning near unshielded syringe
  - Dispose of syringe immediately into shielded container
  - Injection time contributes significantly to extremity dose

1.2.4.4 Key Training Message

Radiopharmaceutical safety is multidimensional: Radiation protection, pharmaceutical sterility, patient identification, workflow precision, and regulatory compliance. Effective integration of these components ensures both occupational safety and patient safety in nuclear medicine practice.

1.2.5 Contamination Monitoring (GM Survey, Wipe Testing)

Contamination monitoring is essential in nuclear medicine due to the routine handling of unsealed radioactive sources. Effective monitoring prevents spread of radioactive material, reduces internal exposure risk, and ensures compliance with regulatory and institutional safety standards.

1.2.5.1 Types of Contamination

- Removable (Loose) Contamination
  - Can be transferred by contact
  - Detectable by wipe testing
  - Represents inhalation or ingestion risk
- Fixed Contamination
  - Adhered to surfaces
  - Detected by direct survey
  - Lower transfer risk but still requires documentation

1.2.5.2 GM Survey Monitoring (Direct Surface Survey)

- Instrumentation
  - Geiger–Müller (GM) survey meter
  - Thin-window probe preferred for low-energy gamma and beta emitters
  - Calibrated annually

  - Battery and background check before use
- Survey Procedure
  - Perform background measurement
  - Hold probe ~1–2 cm from surface
  - Move slowly (~2–5 cm/sec)
  - Monitor audible count rate
  - Focus on high-risk areas: Hot lab benches, L-block surfaces, Injection trays, Waste containers, Door handles, Floors near preparation area
- Interpretation
  - Compare readings to background
  - Investigate readings >2–3× background
  - Document elevated findings
  - Decontaminate and re-survey

1.2.5.3 Wipe Testing (Removable Contamination Assessment)

Purpose: Wipe testing assesses removable contamination and internal exposure risk.

- Materials
  - Filter paper or cotton swab
  - Defined surface area (~100 cm^2)
  - Liquid scintillation counter or gamma counter
- Procedure
  - Wipe defined area using consistent pressure
  - Label sample with location and date
  - Measure in counting system
  - Subtract background
  - Convert counts per minute (CPM) to activity (Bq) using efficiency factor
- Regulatory Guidance: Although limits vary by jurisdiction, common investigation levels are:
  - 3.7 Bq/cm^2 (removable contamination) for gamma emitters
  - Department-specific internal thresholds recommended
  - Any value above action level requires decontamination and repeat testing
- Monitoring Frequency
  - Routine Monitoring: Daily in hot lab, weekly in controlled areas, after each radiopharmaceutical preparation session, immediately following any spill
  - Event-Based Monitoring: After contamination alarm, following suspected glove tear, after unusual dose reading
- Personal Contamination Monitoring
  - Before leaving controlled area: Survey hands, survey shoes, survey lab coat, check extremity dosimeter area
  - Contaminated PPE must be removed and controlled
- Decontamination Procedure: If contamination detected:
  - Isolate area
  - Wear appropriate PPE
  - Use absorbent material
  - Clean from outer edge inward
  - Re-survey

    - Document results
    - Repeated cleaning may be required.
- Documentation and Quality Assurance: All monitoring activities should be:
    - Logged with date/time
    - Linked to operator
    - Reviewed by Radiation Safety Officer
    - Included in routine safety audit
    - Trend analysis can identify workflow weaknesses

1.2.5.4 Key Training Message

Contamination monitoring is not merely regulatory compliance — it is an active barrier against internal exposure and environmental spread. Proper GM survey technique, systematic wipe testing, clear action thresholds, and documentation are essential components of a safe nuclear medicine program.

1.2.6 Spill Management and Emergency Response Procedures

Spill management and emergency response procedures are critical components of radiation protection in nuclear medicine, particularly in facilities handling unsealed radionuclides. Prompt, structured response minimizes contamination spread, reduces occupational exposure, and ensures regulatory compliance.

1.2.6.1 Classification of Spills

Spills should be categorized to guide response intensity

- Minor Spill
    - Small volume
    - Limited to controlled area
    - No personal contamination
    - Low activity level
    - No airborne risk
- Major Spill
    - Large volume or high activity
    - Spread beyond immediate work surface
    - Personal contamination suspected
    - Potential inhalation risk
    - Occurs outside designated controlled area

1.2.6.2 Immediate Response Actions

- Step 1: Stop and Secure
    - Cease work immediately
    - Warn nearby personnel
    - Prevent spread (restrict movement)
- Step 2: Personal Safety
    - Wear appropriate PPE (gloves, lab coat, shoe covers)

  - Avoid direct contact
  - Monitor hands if contamination suspected

1.2.6.3 Minor Spill Procedure

- Restrict access to area
- Cover spill with absorbent material
- Work from outer edge inward
- Collect contaminated materials into radioactive waste
- Survey area using GM meter
- Perform wipe testing if required
- Repeat decontamination until levels approach background
- Document incident
- Minor spills must still be logged and reviewed by the Radiation Safety Officer (RSO)

1.2.6.4 Major Spill Procedure

- Evacuate non-essential personnel
- Prevent contamination spread (block area)
- Notify RSO immediately
- Remove contaminated clothing if necessary
- Assess personal contamination
- Implement systematic decontamination
- Perform contamination survey and wipe tests
- Document and file incident report
- Airborne release risk requires respiratory protection and possible room ventilation assessment

1.2.6.5 Personal Contamination Management

- If skin contamination occurs: Remove contaminated clothing, Wash gently with lukewarm water and mild soap, avoid abrasive scrubbing, re-survey, repeat until acceptable levels achieved
- If internal contamination suspected: Notify RSO and physician, consider bioassay, document exposure

1.2.6.6 Spill Kit Contents

Each controlled area should maintain a spill kit containing: Absorbent pads, disposable gloves, shoe covers, plastic waste bags, warning signage, decontamination solution, wipe test materials, incident report forms

1.2.6.7 Documentation Requirements

All spills must be: Recorded with date/time, identified by radionuclide and estimated activity, described in terms of location and cause, reviewed by RSO, included in QA records, root cause analysis is recommended for major events.

1.2.6.8 Post-Incident Review

After spill resolution: Review workflow, identify contributing factors, update SOP if needed, conduct refresher training, monitor involved personnel dose records.

1.2.6.9 Integration with ALARA

Effective spill response reduces: Secondary contamination, extremity dose accumulation, internal exposure risk, psychological stress among staff, preparedness strengthens overall radiation safety culture.

1.2.6.10 Key Training Message

Spill management is a controlled, structured process—not a panic response. Clear classification, immediate containment, contamination monitoring, documentation, and supervisory oversight are essential to maintaining safe nuclear medicine operations.

1.2.7 Radioactive Waste Segregation and Decay-In-Storage Procedures

Radioactive waste management in nuclear medicine must ensure safe handling, regulatory compliance, contamination prevention, and dose optimization. Because most diagnostic radionuclides used in PET/CT and conventional nuclear medicine are short-lived, decay-in-storage (DIS) is the primary disposal strategy.

1.2.7.1 Classification of Radioactive Waste

Radioactive waste in nuclear medicine is typically categorized by:

- Physical Form
    - Solid (syringes, gloves, absorbent pads, tubing)
    - Liquid (residual radiopharmaceutical solutions)
    - Sharps (needles, broken glass)
- Radionuclide Half-Life
    - Short-lived radionuclides (e.g., ^{99m}Tc, ^{18}F, ^{131}I diagnostic quantities) are suitable for decay-in-storage.
    - Long-lived radionuclides require licensed disposal pathways.

1.2.7.2 Waste Segregation Principles

Segregation must occur at the point of generation. Waste should be separated by: Radionuclide, half-life category, physical form, sharps vs non-sharps, contaminated vs non-contaminated materials, mixing radionuclides complicates decay calculations and regulatory release.

1.2.7.3 Decay-in-Storage (DIS) Procedures

- Applicability: DIS is appropriate for radionuclides with half-lives generally less than 120 days (jurisdiction-dependent), commonly: ^{99m}Tc (6 hours), ^{18}F (110 minutes), ^{131}I diagnostic doses, and ^{123}I

- Storage Requirements: Shielded container, clearly labeled with radionuclide and date, secured within controlled area, separated by half-life group, logged in waste inventory record
- Decay Rule: Waste must be stored for a minimum of 10 half-lives to ensure activity decreases to background levels. Example:
  - ^{18}F (110 min half-life) – 10 half-lives ≈ 18 hours
  - ^{99m}Tc (6 hours) – 10 half-lives ≈ 60 hours
- Release Verification: Before disposal as non-radioactive waste:
  - Remove shielding
  - Survey with GM meter
  - Confirm radiation indistinguishable from background
  - Remove radiation labels
  - Deface radioactive markings and Document release
- Liquid Waste Management: Short-lived liquid waste may be:
  - Held for decay
  - Discharged according to regulatory limits
  - Logged and documented
  - Liquid waste disposal must comply with national discharge regulations
- Sharps Disposal: Radioactive sharps must:
  - Be placed in puncture-resistant containers
  - Be segregated by radionuclide
  - Undergo decay-in-storage before final disposal
  - Have labels removed after survey clearance
- Documentation and Record Keeping: Waste records must include:
  - Radionuclide
  - Estimated activity
  - Date of generation
  - Storage start date
  - Survey date prior to release
  - Name of responsible personnel
  - Records should be retained according to regulatory requirements
- Integration with Radiation Protection: Proper waste segregation:
  - Reduces contamination risk
  - Minimizes extremity exposure
  - Supports regulatory compliance
  - Enhances safety culture
  - Prevents environmental release

1.2.7.4 Key Training Message

Radioactive waste management in nuclear medicine is primarily a time-based control strategy. Proper segregation, accurate labeling, adequate decay storage, systematic survey verification, and documentation are essential to maintain safe and compliant practice.

1.3 Evaluation Methodology

Program effectiveness was evaluated using structured pre- and post-training assessments measuring radiation safety knowledge and self-reported protocol adherence. Compliance was additionally assessed using structured observational checklists evaluating:

- Proper use of extremity dosimeters.
- Appropriate shielding application.
- Adherence to standardized handling procedures.
- Contamination monitoring practices.
- Spill response preparedness.

Pre–post differences were analyzed using non-parametric statistical methods. Quantitative outcomes are presented in Figure 1B of the main manuscript, and the structured performance checklist is provided in Table S1.

1.3.1 Scoring Method

Each checklist item was scored using a three-point scale:

- 2 = Fully Competent
- 1 = Partially Competent / Minor Deviation
- 0 = Non-Compliant / Incorrect / Not Demonstrated

The Total Score was calculated as the sum of all item scores, with a maximum possible score of 42. The Compliance Percentage was calculated as:

$$\text{Compliance Percentage } = \frac{\text{Total Score}}{42} \times 100$$

1.3.2 Interpretation of Compliance Level

- ≥ 85% = Competent for independent practice
- 70–84% = Competent with supervision
- < 70% = Additional training required

1.4 The questionnaire

This questionnaire is part of a research study conducted by the Department of Radiology, Faculty of Medicine, Prince of Songkla University. The study aims to assess occupational radiation exposure risks and factors affecting the quality of life and health safety of nuclear medicine personnel. We respectfully request your cooperation in completing this questionnaire by providing accurate and honest responses based on your experiences and perspectives. All information collected will be used solely for academic research purposes. Responses will be kept strictly confidential, and no personally identifiable information will be recorded. Only the research team will have access to the data, and all results will be reported in aggregate form to ensure anonymity. Your participation is voluntary, and your thoughtful responses are greatly appreciated. We sincerely thank you for your time and valuable contribution to this study.

The assessment of factors affecting the quality of life and health safety of nuclear medicine personnel is structured into:

- Part 1: General information about the respondent
- Part 2: Basic knowledge of radiation and radiation protection

Please select the response that most accurately reflects your situation or opinion. Only one option should be selected for each item. Thank you very much for your cooperation.

1.4.1 Part 1: General Information of the Respondents

Question	Response Options	A
1. Respondent ID	________________________________	A1:
2. Biological sex	☐ Female ☐ Male	A2:
3. Age (years)	☐ ≤20 ☐ 20–29 ☐ 30–39 ☐ 40–49 ☐ 50–59	A3:
4. Education level	☐ High School/Vocational Certificate<br>☐ Associate Degree/Vocational Certificate<br>☐ Bachelor's degree<br>☐ Master's degree or higher	A4:
5. Job position	☐ Radiopharmaceutical preparation staff<br>☐ Nurse<br>☐ Radiologic technologist<br>☐ Assistant Radiologic Technologist	A5:
6. Work experience (years)	☐ <1 ☐ 1–5 ☐ 6–10 ☐ 11–15<br>☐ 15–20 ☐ >20	A6:
7. Marital status	☐ Single ☐ Divorced ☐ Married (no children)<br>☐ Married with children	A7:
8. Smoking status	☐ Non-smoker ☐ 1–5 cigarettes/day<br>☐ 6–10 cigarettes/day ☐ >10 cigarettes/day	A8:
9. Exposure to secondhand smoke	☐ None ☐ 1–5 cigarettes/day<br>☐ 6–10 cigarettes/day ☐ >10 cigarettes/day	A9:
10. Alcohol consumption	☐ None ☐ 1–5 times/month ☐ 5–10 times/month<br>☐ >10 times/month	A10:
11. Exercise frequency	☐ None ☐ 1–2 days/week ☐ 3–4 days/week<br>☐ >4 days/week	A11:
12. Working hours per week	☐ ≤40 ☐ 40–50 ☐ 51–60 ☐ >60	A12:
13. Use of radiation protective measures	☐ Never ☐ Sometimes ☐ Often<br>☐ Almost every time ☐ Every time	A13:
14. Radiation protection training received	☐ Never ☐ Yes (___ years ago)	A14:
15. Radiation Safety Officer (RSO) license status	☐ None ☐ First-level ☐ Mid-level ☐ High-level	A15:

Question	Response Options	A
16. Need for radiation protection training workshop	☐ At least twice/year ☐ At least once/year ☐ Not required	A16:
17. Concern about radiation-related occupational risk	☐ Not worried ☐ Slightly worried ☐ Moderately concerned ☐ Very worried ☐ Extremely anxious	A17:

1.4.2 Part 2: Basic Knowledge of Radiation and Protection from Radiation Hazards

Question	Response Options	Answer Key
1. Severity of injury from external radiation exposure depends on:	☐ Source strength<br>☐ Duration of exposure<br>☐ Source strength and duration of exposure<br>☐ Duration and body strength<br>☐ Don't know	Correct answer: Source strength and duration of exposure
2. For pregnant radiation workers (≤1 mSv total), the average monthly limit is:	☐ 0.1 mSv<br>☐ 0.2 mSv<br>☐ 0.25 mSv<br>☐ 0.5 mSv<br>☐ Don't know	Correct answer: 0.1 mSv
3. The body tissue that changes most rapidly after radiation exposure is:	☐ Bone marrow<br>☐ Digestive tract<br>☐ Skin<br>☐ Reproductive organs<br>☐ Don't know	Correct answer: Bone marrow
4. Effects occurring after low-dose radiation exposure include:	☐ Bone marrow failure within 1 year<br>☐ Immediate nervous system death<br>☐ Cancer and genetic effects<br>☐ No effect<br>☐ Don't know	Correct answer: Cancer and genetic effects
5. Phase 1 (prodromal phase) radiation symptoms include:	☐ No symptoms<br>☐ Fatigue, vomiting, diarrhea, fever<br>☐ Decreased immunity<br>☐ Bleeding tendency<br>☐ Don't know	Correct answer: Fatigue, vomiting, diarrhea, fever
6. Radiation that requires lead shielding is:	☐ Alpha<br>☐ Beta<br>☐ Neutron<br>☐ Gamma<br>☐ Don't know	Correct answer: Gamma
7. Annual occupational dose	☐ 1 mSv	Correct answer: 20 mSv

Question	Response Options	Answer Key
limit averaged over 5 consecutive years is:	☐ 10 mSv<br>☐ 20 mSv<br>☐ 50 mSv<br>☐ Don't know	
8. Controlled area threshold (fraction of 20 mSv/year) is:	☐ 1/4 ☐ 1/3 ☐ 1/2 ☐ 1<br>☐ Don't know	Correct answer: 1/3
9. Patients and workers receive radiation from:	☐ Contamination<br>☐ Ingestion<br>☐ Inhalation<br>☐ All of the above<br>☐ Don't know	Correct answer: All of the above
10. Radiation protection measures for workers include:	☐ ALARA principles<br>☐ Wearing dosimeter<br>☐ Lead vest & thyroid shield<br>☐ All of the above<br>☐ Don't know	Correct answer: All of the above

1.4.3 Radiation Safety Opinion Survey (Likert Scale)

Question	Strongly agree	Agree very much	Moderately agree	Disagree	Strongly disagree
**1. Rules and regulations regarding safety**					
1.1 You have received advice on measures to prevent radiation hazards in the organization.	☐	☐	☐	☐	☐
1.2 You see details such as the name and contact number of the Radiation Protection Officer (RPO) in the organization.	☐	☐	☐	☐	☐
1.3 You found relevant information about radiation dose limits, regulations, and safety measures at the organization.	☐	☐	☐	☐	☐
1.4 You found that all staff in the organization comply with radiation protection measures and safety regulations.	☐	☐	☐	☐	☐
1.5 You are confident in your knowledge and understanding of radiation protection measures and safety regulations.	☐	☐	☐	☐	☐
**2. Equipment related to radiation protection**					

Question	Strongly agree	Agree very much	Moderately agree	Disagree	Strongly disagree
2.1 Personal radiation measurement equipment at service agencies is sufficient and usable.	☐	☐	☐	☐	☐
2.2 You wear a device to measure your radiation exposure at all times while working.	☐	☐	☐	☐	☐
2.3 You receive reports and records of personal radiation levels regularly during each reporting period.	☐	☐	☐	☐	☐
**3. Radiation protection materials**					
3.1 The design of your organization follows international standards.	☐	☐	☐	☐	☐
3.2 Relevant officials regularly measure radiation in the work area.	☐	☐	☐	☐	☐
3.3 The agency has appropriate and sufficient radiation protection equipment (lead screens, lead vests, thyroid shields, syringe shields, lead containers, protective glasses, etc.).	☐	☐	☐	☐	☐
3.4 The organization has sufficient materials and safeguards to protect against patient-emitted radiation.	☐	☐	☐	☐	☐
3.5 You regularly use personal radiation protection equipment while working.	☐	☐	☐	☐	☐

2 Supplementary Table

Table S1: Radiation Safety Competency Assessment Checklist (Post-Training Evaluation). Each competency domain was assessed as:

C = Competent | NI = Needs Improvement | NA = Not Applicable.

Domain	Competency Indicator	Observable Assessment Criteria	C 2	NI 1	NA 0	Comments
Radiation Protection Principles	Explains Justification principle	Clearly articulates clinical justification and avoidance of unnecessary exposure				
	Explains Optimization (ALARA)	Describes time–distance–shielding strategies with practical examples				
	Explains Dose Limitation	Correctly states occupational and public dose limits				
Occupational Dose Limits Knowledge	Identifies whole-body dose limits	Correctly states 20 mSv/year averaged over 5 years				
	Identifies extremity dose limits	Correctly states 500 mSv/year				
	Identifies lens dose limits	Correctly states 20 mSv/year				
Dosimetry Compliance	Proper whole-body dosimeter use	Worn at correct position and not shared				
	Proper extremity dosimeter placement	Positioned on dominant hand at appropriate location				
	Reviews personal dose reports	Demonstrates understanding of monthly dose summary				
Time Optimization	Minimizes handling time	Efficient radiopharmaceutical preparation without unnecessary delay				
	Avoids unnecessary proximity	Maintains distance when clinically feasible				
Shielding Practices	Uses syringe shields	Applies correct shielding for radionuclide energy				

Domain	Competency Indicator	Observable Assessment Criteria	C 2	NI 1	NA 0	Comments
	Uses L-block/vial pigs appropriately	Ensures shielding barrier placement during preparation				
Radiopharmaceutical Handling	Follows standardized workflow	Adheres to SOP for preparation and administration				
	Prevents contamination	Maintains clean working surfaces and uses absorbent pads				
Contamination Monitoring	Performs GM survey correctly	Demonstrates correct technique and interpretation				
	Conducts wipe testing	Demonstrates proper sample collection and documentation				
Emergency Preparedness	Demonstrates spill response	Follows institutional spill protocol correctly				
	Reports incidents appropriately	Notifies RSO and documents event per policy				
Safety Culture & Governance	Participates in safety reporting	Demonstrates understanding of non-punitive reporting system				
	Adheres to ALARA mindset	Consistently applies exposure minimization strategies				

Overall Rating:
☐ Competent for independent practice
☐ Competent with supervision
☐ Additional training required

Evaluator: ____________________________
Date: ________________________________

3 Supplementary Figures

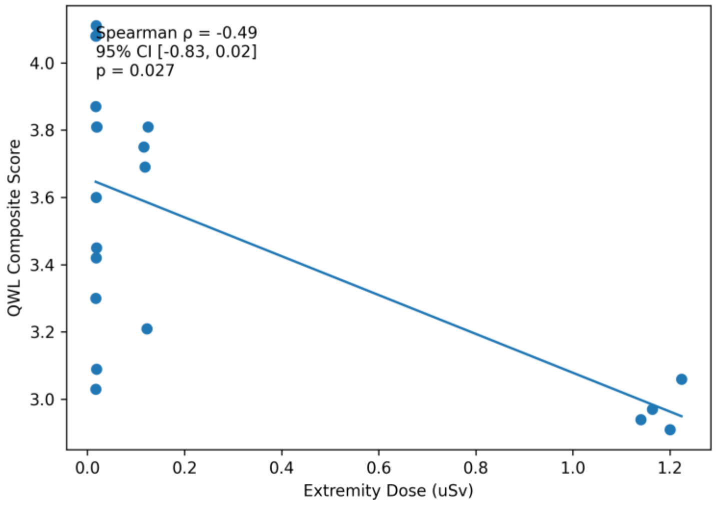

Figure S1. Association between extremity radiation dose (μSv) and QWL composite score (n = 20). Scatter plot with fitted linear trend line for visualization. Spearman's rank correlation demonstrated a moderate inverse association (ρ = −0.49, 95% CI −0.83 to 0.02, p = 0.027). Extremity dose reflects dominant-hand mean exposure (thumb, index finger, palm)

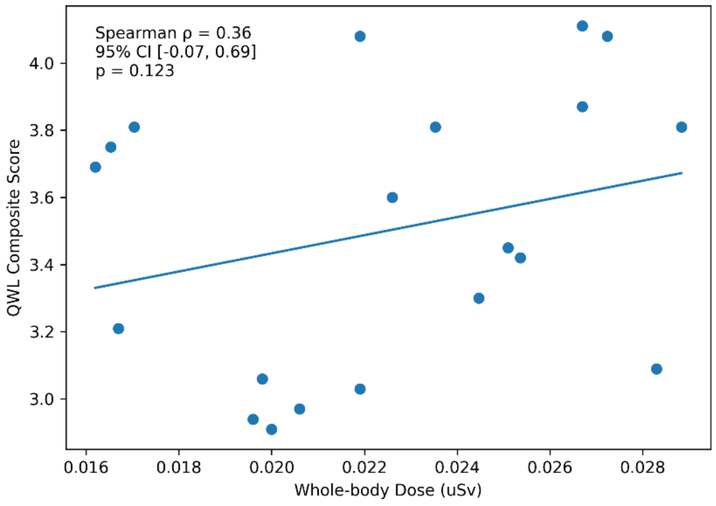

Figure S2. Association between whole-body radiation dose (μSv) and QWL composite score (n = 20). Scatter plot with fitted linear trend line shown for visualization. Spearman's rank correlation demonstrated a non-significant positive association (ρ = 0.36, 95% CI −0.07 to 0.69, p = 0.123). Whole-body dose reflects personal dosimeter measurements recorded during routine occupational monitoring.

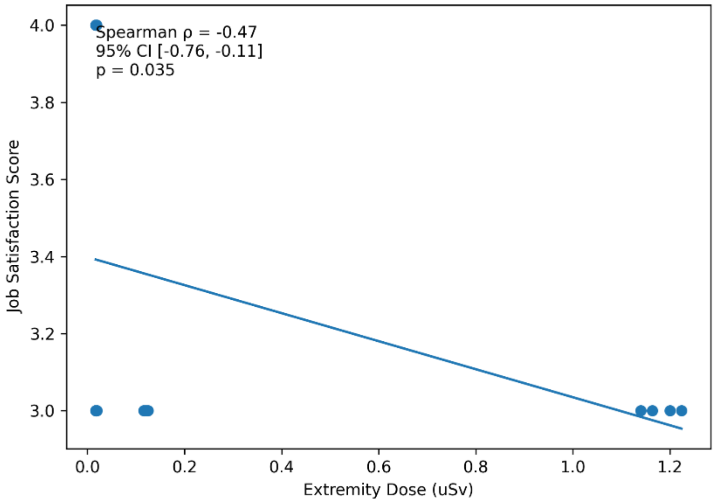

Figure S3. Association between extremity radiation dose (µSv) and job satisfaction score (n = 20). Scatter plot with fitted linear trend line shown for visualization. Spearman's rank correlation demonstrated a moderate inverse association (ρ = −0.47, 95% CI −0.76 to −0.11, p = 0.035), indicating lower job satisfaction scores with increasing extremity exposure. Extremity dose reflects dominant-hand mean exposure measured during routine occupational monitoring.

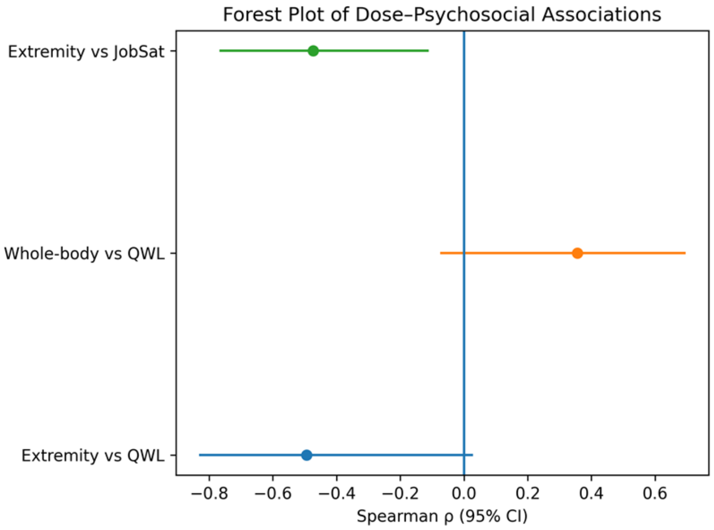

Figure S4. Forest plot summarizing associations between radiation dose metrics and psychosocial outcomes (n = 20). Points represent Spearman's rank correlation coefficients (ρ), and horizontal lines denote 95% confidence intervals. Extremity dose demonstrated moderate inverse associations with QWL and job satisfaction, whereas whole-body dose showed a positive but non-significant association with QWL. The vertical reference line at ρ = 0 indicates no correlation. Associations crossing the null line are not statistically significant at α = 0.05.

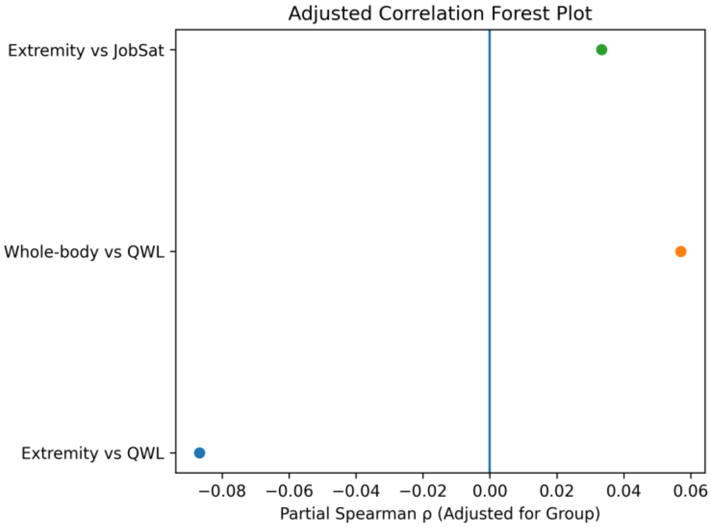

Figure S5. Adjusted correlation forest plot showing partial Spearman correlation coefficients (ρ) between radiation dose metrics and psychosocial outcomes after controlling for occupational group (n = 20). Points represent partial correlation estimates adjusted for professional role. Following adjustment, associations between extremity dose and both QWL and job satisfaction were attenuated toward the null, and the association between whole-body dose and QWL remained minimal. The vertical reference line at ρ = 0 indicates no association. These results suggest that unadjusted correlations were largely influenced by occupational clustering.
